# Advancing mental health and well-being of Nigerian children through public health screening for Fragile X disorders (CHAMP-FX): protocol of a prospective multicentre screening study with longitudinal follow-up

**DOI:** 10.1371/journal.pone.0355384

**Published:** 2026-08-05

**Authors:** Chioma Ngozichukwu Pauline Mbachu, Edwin Ehi Eseigbe, Ikechukwu Innocent Mbachu, George Uchenna Eleje, Ifeoma Bridget Udigwe, Chika Onwuwamah, Ifeoma Chisom Iloghalu, Justus Onu, Onochie Patrick Manafa, Onyekachi Okonkwo, Halima Adamu, Rasaki Aliu, Chizalu Ifeyinwa Ndukwu, Victor Ugochukwu Ndubuisi, Theresa Erezimena Ogholaja, Samuel Okwuchukwu Ilikannu, Ahoma Mbanuzuru, Kingsley Anukam, Gbenga Olorunfemi, Amalachukwu Okwukweka Odita, Divinefavour Malachy Echezona, Uzoma Okereke, Esther Umeadi, Gerald Okanandu Udigwe, Amarachi Ujunwa Bayo, Jacinta Elo-Ilo, Maria-Regina Idume, Chijioke Elias Ezeudu, Kelechi Odinaka, Zita Chidera Ezeno, Binyelum Ofojebe, Adaeze Obiegbu, Chukwudubem Onyejiaka, Amara Egbogu, Obed Chukwuma Azubike, Ngozi Chinenye Okeke, Esther Nwabuife Ugochukwu, Sunday Onyemaechi Oriji, Yves-Ann Amaka Ezeuko, Udoka Peace Adeniyi, Lazarus Ugochukwu Okafor, Joy Chinelo Ebenebe, Uwakwe Richard, Clement Chukwudorue Ezechukwu, Flora Tassone, Randi Hagerman

**Affiliations:** 1 Department of Paediatrics, Nnamdi Azikiwe University and Teaching Hospital, Awka, Anambra, Nigeria; 2 Department of Paediatrics, Benue State University, Makurdi, Benue, Nigeria; 3 Department of Obstetrics and Gynaecology, Nnamdi Azikiwe University and Teaching Hospital, Awka, Anambra, Nigeria; 4 Department of Community Medicine, Nnamdi Azikiwe University and Teaching Hospital, Awka, Anambra, Nigeria; 5 Centre for Human Virology and Genomics, Microbiology Department, Nigerian Institute of Medical Research, Lagos, Nigeria; 6 Division of Preventive Medicine, Department of Medicine, University of Alberta, Edmonton, Alberta, Canada; 7 Department of Mental Health, Nnamdi Azikiwe University and Teaching Hospital, Awka, Anambra, Nigeria; 8 Department of Clinical Chemistry, Nnamdi Azikiwe University, Awka, Anambra, Nigeria; 9 Department of Clinical Psychology, Nnamdi Azikiwe University, Awka, Anambra, Nigeria; 10 Department of Paediatrics, Aminu Kano Teaching Hospital, Kano, Nigeria; 11 Department of Paediatrics, Gombe State University, Gombe, Gombe, Nigeria; 12 Department of Paediatrics, Federal Medical Centre Asaba, Delta, Nigeria; 13 Department of Obstetrics and Gynaecology, Federal Medical Centre Asaba, Delta, Nigeria; 14 Department of Medical Microbiology & Public Health, Nnamdi Azikiwe University, Awka, Anambra, Nigeria; 15 Department of Research & Development, Uzobiogene Genomics, London, Ontario, Canada; 16 Department of Paediatrics, Federal Medical Centre, Ebute Metta, Lagos, Nigeria; 17 Department of Nursing Services, Nnamdi Azikiwe University and Teaching Hospital, Awka, Anambra, Nigeria; 18 Department of Paediatrics, Federal University Teaching Hospital, Owerri, Imo State, Nigeria; 19 Department of Physiotherapy, Nnamdi Azikiwe University and Teaching Hospital, Awka, Anambra, Nigeria; 20 Department of Sociology/Social Demography, Nnamdi Azikiwe University, Awka, Anambra, Nigeria; 21 Department of Surgery, Nnamdi Azikiwe University and Teaching Hospital, Awka, Anambra, Nigeria; 22 Department of Pharmacy and Pharmacovigilance, Nnamdi Azikiwe University and Teaching Hospital, Awka, Anambra, Nigeria; 23 Department of Paediatrics, Olabisi Onabanjo University Teaching Hospital, Sagamu, Ogun, Nigeria; 24 MIND Institute, University of California Davis, Sacramento, California, United States of America; University of Rwanda College of Medicine and Health Sciences, RWANDA

## Abstract

There is an increasing burden of neurodevelopmental disorders worldwide, with scarce data in low and middle-income countries, including Nigeria. Little is known about Fragile X disorders (Fragile X syndrome and Fragile X premutation-associated conditions) in developing countries and there is no national data in Nigeria. Advancing Mental Health and Well-being of Nigerian Children Through Public Health Screening for Fragile X Disorders (CHAMP-FX) is a multicentre public health screening initiative designed to estimate the prevalence of Fragile X disorders among children with neurodevelopmental disorders in Nigeria through targeted screening, strengthen diagnostic capacity and provide pathways to targeted treatments. This is a prospective multicentre screening study with longitudinal follow-up recruiting children aged 1–18 years with intellectual disability, autism spectrum disorder, and/or global developmental delay from six tertiary hospitals across Nigeria’s six geopolitical zones. Using purposive sampling, 102 participants (17 per zone) will be enrolled. Sociodemographic data and clinical evaluation will be obtained using KoboToolbox. Blood samples will be collected as dried spots on quick-response coded filter cards, stored with desiccant, and transported to the coordinating molecular laboratory. Genetic testing will be performed using a long-range amplification workflow followed by long-read sequencing to determine repeat sizes and classify results using internationally accepted thresholds. The primary outcome is the proportion of participants with Fragile X full mutation and/or premutation in the selected cohort. Secondary outcomes include the distribution of repeat sizes and associations with sociodemographic and clinical variables. Screen-positive participants will receive structured result disclosure, genetic counselling, and referral for appropriate supportive interventions, with targeted therapy using metformin offered to participants diagnosed with Fragile X syndrome. Findings will be disseminated through peer-reviewed publication, conferences and stakeholder engagement.

## Introduction

Neurodevelopmental disorders such as intellectual disability, global developmental delay, and autism significantly contribute to lifelong disability and mental health challenges globally [1].

Fragile X syndrome (FXS) is the leading inherited cause of intellectual disability and a major single-gene contributor to autism, resulting from cytosine-guanine-guanine (CGG) trinucleotide repeat expansion (>200 repeats) in the 5′ untranslated region (5’UTR) of the fragile X mental retardation 1 (*FMR1)* gene, resulting in gene silencing and reduced/absent fragile X mental retardation protein (FMRP) and subsequent synaptic dysfunction [2–4].

Beyond full mutation FXS, individuals carrying *FMR1* premutation alleles (approximately 55–200 CGG repeats) are at risk of Fragile X–associated premutation conditions, including Fragile X–associated primary ovarian insufficiency (FXPOI), Fragile X–associated tremor/ataxia syndrome (FXTAS), and Fragile X–associated neuropsychiatric disorders (FXAND), thereby extending the clinical and psychosocial burden of *FMR1*-related disorders also known as Fragile X disorders across the lifespan [4,5].

There have been significant global advances in diagnosis and targeted supports, in Nigeria, Fragile X disorders are rarely identified in routine clinical practice, mainly due to low awareness, limited access to molecular diagnostics, and persistent sociocultural stigma surrounding neurodevelopmental conditions [1,5–7] This implies that affected children and families experience delays in management, while policymakers and clinicians lack the data required for informed service planning.

The World Health Organisation has emphasised early identification and intervention for developmental challenges in low and middle-income countries (LMICs) and highlighted neglected needs of children with developmental disabilities [8]. CHAMP-FX aligns with these priorities and conducts a multi-site screening approach in high-risk paediatric populations, thereby strengthening local capacity for Fragile X testing and follow-up.

## Objectives

Overall aim: To advance Nigerian children’s mental health and well-being through a public health screening initiative for Fragile X disorders (CHAMP-FX).

### Specific objectives

1. To estimate the prevalence of Fragile X full mutation and premutation among Nigerian children (1–18 years) with intellectual disability, autism spectrum disorder, or global developmental delay using molecular methods.
2. To identify sociodemographic and clinical factors associated with Fragile X disorders in this population.
3. To establish screening-to-care pathways, including result disclosure, genetic counselling, and referral for supportive interventions.
4. To develop a context-appropriate model for integrating Fragile X screening into paediatric/developmental health services in Nigeria.

## Materials and methods

### Study Setting and Study design

Nigeria is a country in West Africa with an estimated 2024 total population of 232,679,478 [9]. The country comprises six geopolitical zones (North-central, north-east, north-west, south-east, south-south and south-west), each with distinct sociocultural and healthcare characteristics. CHAMP-FX is a prospective multicentre screening study with longitudinal follow-up to be conducted across six tertiary hospitals representing Nigeria’s six geopolitical zones. This approach enables the study to track outcomes over time, assess longitudinal screening outcomes, referral processes, and implementation feasibility.

The coordinating centre is Nnamdi Azikiwe University/Teaching Hospital, Nnewi, Anambra State, south-east Nigeria. The study sites will include Nnamdi Azikiwe University/Teaching Hospital, Anambra (Coordinating centre)- (Southeast), Benue State University Teaching Hospital, Benue (Northcentral), Federal Medical Centre, Asaba, Delta (Southsouth), Aminu Kano Teaching Hospital, Kano (Northwest), Federal Teaching Hospital, Gombe (Northeast), and Federal Medical Centre, Ebute-Metta, Lagos (Southwest).

### Study population

Children aged 1–18 years attending paediatric neurology/paediatric clinics with a diagnosis of: intellectual disability, autism spectrum disorder, and/or global developmental delay. Autism spectrum disorder, intellectual disability and global developmental delay were confirmed using the DSM-5 diagnostic criteria through multidisciplinary assessments conducted by a trained neurodevelopmental paediatrician, paediatric neurologists and a psychologist. Autism spectrum disorder was diagnosed based on developmental history, clinical observation and behavioural assessment. For children younger than 4 years, the Ages and Stages Questionnaire, Third Edition (ASQ-3), was used to assess developmental delay. For children aged 4 years and older, the Raven’s Standard Progressive Matrices (Revised 2019) was used to assess intellectual functioning as part of the evaluation for intellectual disability.

### Study period

The study is scheduled to run from October 2025 to September 2027, following fund disbursement. Participant recruitment is planned between February and September 2026. Data collection is expected to be completed by January 2027, with analysis and results anticipated by May 2027. Follow-up will be conducted across the six sites. These timelines are projected estimates and may be adjusted in response to operational, logistical, or field conditions. (S1 Table)

### Eligibility criteria

Inclusion: Age 1–18 years, clinical diagnosis of intellectual disability, autism spectrum disorder, or global developmental delay; and parent/guardian provides written informed consent (and child assent where appropriate). Children whose parent/guardian declines consent will be excluded. See Appendix A

### Sample size and allocation

The minimum sample size was calculated using the formula [10]: Z2Pq/d2 n = unadjusted minimum sample size, Z = 1.96, P = 0.023 [11], q = 1-P = 0.977, d = standard error (5%, 0.05). Therefore, $n=\frac{\text{1}.\text{96 x }\text{1}.\text{96 x }\text{0}.\text{023 x }\text{0}.\text{977}}{\text{0}.\text{05 x }\text{0}.\text{05}}$ = 34.5. To adjust for a finite population, the formula $nc=\frac{\text{n}}{\text{1}+(\frac{\text{n}}{\text{N}})}\;$will be used, where nc is adjusted minimum sample size, N = Population from the record (from the records, 12 cases of autism are seen every 5 months in NAUTH, Nnewi, Anambra State. This amounts to 28.9 patients every year). $nc=\frac{\text{34}.\text{5}}{\text{1}+(\frac{\text{34}.\text{5}}{\text{28}.\text{9}})}$ = 15.7 (minimum sample size). For the purpose of this study, 17 participants will be selected per geo-political zone, yielding 102 study participants. Due to the absence of national prevalence data on Fragile X syndrome among high-risk children in Nigeria, the sample size was estimated using patient records from the coordinating centre, a major referral centre for neurodevelopmental disorders. These represented the best available local data to inform the study design. While this may not fully capture regional variation, the multicentre design will enhance the representativeness of the study findings.

### Sampling technique

Purposive sampling of eligible clinic attendees will be used at each site until the zone allocation is achieved. To ensure national representation and account for regional variability: Equal allocation of participants was adopted, with 17 children per zone, resulting in a total of 102 participants. This pragmatic sample is intended to estimate prevalence estimates within this high-risk clinical cohort and support exploratory analyses of correlates.

### Data collection

A structured interviewer-administered proforma will capture – Child: age, sex, developmental history, clinical diagnosis, schooling status; Parent/guardian: age, education, occupation, household socioeconomic indicators, and family history.(S1 Appendix)Clinical assessment will include vitals, anthropometry, general examination and documentation of Fragile X-associated clinical features using established checklists [5,12].

### Specimen collection and handling (dried blood spots)

Dried blood spots (DBS) will be collected on Quick Response (QR-coded) and labelled Whatman 903 Protein Saver Cards (Cytiva MA, USA) following standard DBS procedures, including universal precautions [13, 14] First, the finger will be pricked using a sterile lancet after alcohol cleansing. Then, blood will be applied to card circles; air dried horizontally for ~3 hours and each card stored in an individual pouch with a desiccant; labelled using QR-coded identification only and stored for a short term in a refrigerator at 2–8°C at zonal sites before batch transport. Samples will then be transported in batches using a dedicated medical courier service that complies with applicable biospecimen transport requirements to the coordinating molecular laboratory in Nnamdi Azikiwe University, Anambra State, Nigeria.

### Laboratory workflow

#### DNA extraction and quality assessment.

Genomic DNA will be extracted from dried blood spot (DBS) specimens at the coordinating molecular laboratory using a validated DNA extraction workflow optimised for *FMR1* molecular analysis and performed according to established standard operating procedures. The workflow includes specimen receipt and verification, DNA extraction, DNA quantification using a validated fluorometric method, and quality assessment to ensure adequate DNA yield and quality for long-range PCR before amplification. Samples with inadequate DNA yield or quality, or those that fail initial amplification, will undergo repeat extraction and repeat testing where sufficient specimen is available. As part of external quality assurance, a subset of samples will undergo repeat testing at the University of California, Davis molecular laboratory (UCD) to verify the accuracy and reproducibility of the molecular analyses [15].

#### Long-range PCR for FMR1 CGG-repeat region.

Long-range PCR will target the *FMR1* 5′UTR CGG repeat using established primer strategies described in Fragile X molecular workflows [13,15,16]. Amplification will be performed using Expand Long Template PCR System (Roche Diagnostics) or equivalent long-range, GC-rich buffer master-mix polymerase conditions, consistent with published Fragile X mutation detection methods and GC-enhancer use (e.g., betaine) for this locus [17–19]. Each run will include a No-template control (NTC) and Positive control DNA with known CGG repeat sizes (sourced/verified through established Fragile X reference laboratories, such as the UCD Molecular Laboratory, Sacramento under the direction of Dr. Flora Tassone) PCR products will be checked by agarose gel electrophoresis consistent with long-range Fragile X assays [17].

#### Amplicon purification and library preparation.

Successful amplicons will be purified (magnetic beads). Purified products will be quality-checked for concentration/integrity and library preparation done using the manufacturer-recommended required input appropriate for the amplicon size [20].

#### Nanopore sequencing and bioinformatics.

Amplicons will be prepared using Oxford Nanopore ligation sequencing chemistry with native barcoding, sequenced on R10.4.1 MinION Mk1D flow cells following Oxford Nanopore Technologies (ONT) guidance [21,22]. Reads will be aligned to a human reference genome using an established long-read aligner. *FMR1*-aligned reads will be analysed using long-read repeat tools to estimate per-read CGG repeat counts and mosaicism where present [23]. Alleles will be classified using internationally accepted thresholds (normal, intermediate, premutation, full mutation) as summarised in GeneReviews for *FMR1* disorders [24]. (S2 Appendix)

### Outcomes

The primary outcome is the proportion of participants with *FMR1* full mutation and/or premutation (those who test positive from the screening) in the selected cohort. Secondary outcomes include the distribution of CGG repeat sizes; the association of FMR1 expansion status with sociodemographic and clinical variables; the proportion of screen-positive families receiving counselling, referrals, and linkage to supportive interventions relevant to child mental health and developmental wellbeing; uptake of cascade testing offers; and building research capacity through mentoring early-career faculty members towards training in public health genetics. The study findings will be reported in accordance with the Strengthening the Reporting of Observational Studies in Epidemiology (STROBE) Statement.

### Data management and confidentiality

Participants will be assigned unique study IDs-QR codes; no names will appear on lab tubes/cards. Hard-copy records will be stored in locked cabinets, and electronic data will be housed in password-secured databases with role-based access. Identifiable and genetic data will be stored in password-protected databases with restricted access to authorised study personnel. Data transfers will be conducted using encrypted devices or secure electronic transfer systems in accordance with institutional data protection policies. Only authorised study personnel will access identifiable data.

### Statistical analysis plan

Data will be analysed using STATA version 16 (StataCorp LLC, College Station, TX, USA). Descriptive statistics will be performed using means/Standard deviation or medians/Inter-quartile range for continuous variables. Frequencies/percentages will be calculated for categorical variables. Prevalence estimates will be at 95% confidence intervals while Chi-square or Fisher’s exact tests used for categorical variables where appropriate to test for associations. Logistic regression analyses will be performed to identify factors associated with Fragile X positivity if the number of positive cases is sufficient to support reliable multivariable modelling. Where the number of positive cases is insufficient, analyses will be limited to descriptive and bivariate methods.

Statistical significance will be set at p < 0.05.

We will minimise potential sources of bias through recruitment across Nigeria’s six geopolitical zones, standardised eligibility criteria and data collection procedures, centralised laboratory analysis using validated protocols, and prespecified statistical analyses.

## Result disclosure, counselling, and follow-up

As part of the research protocol, parents, legal guardians or caregivers of children diagnosed with Fragile X syndrome through the study will receive the test results through post-test genetic counselling. Families will be counselled on the implications of the diagnosis and, where appropriate, reproductive counselling will be provided for carriers. Participants will be referred to the trained paediatric neurologists at the participating centres for appropriate clinical evaluation, follow-up and routine management, with referral to the coordinating centre at Nnamdi Azikiwe University Teaching Hospital (NAUTH) where specialist input is required. Referral to appropriate specialists and supportive therapies will also be done as appropriate. Any treatment, including consideration of targeted therapies such as metformin where clinically indicated, will be provided as part of routine clinical care and is outside the scope of this research protocol.

Evidence on treatment approaches, including metformin has been discussed in the Fragile X treatment literature; however, CHAMP-FX is not designed as a randomised treatment trial [25,26]. Relatives of participants who test positive will have cascade testing for relatives (where feasible).

The methodology for developing the integration model will involve stakeholder engagement with the Child Neurology Society of Nigeria, the Paediatric Association of Nigeria, the Federal Ministry of Health, selected State Ministries of Health, the National Primary Health Care Development Agency, and other relevant stakeholders from the public and private health sectors. Findings from the study will be presented to stakeholders and discussed to identify practical approaches for integrating Fragile X screening into existing paediatric and developmental health services in Nigeria. Data generated from the study, together with recommendations from the stakeholder engagement, will be synthesised to develop a context-appropriate Fragile X screening integration model for Nigeria.

### Ethical considerations

Ethical approval was obtained from the National Health Research Ethics Committee of Nigeria (NHREC)-NHREC Approval Number NHREC/01/01/2007-07/04/2025, and concordance from participating institutions. Written informed consent will be obtained from parents or legal guardians, and assent, where feasible, will be sought from children aged seven years and above using age-appropriate explanations, recognising the special needs of children with autism, intellectual disability, or developmental delay. To ensure understanding and ethical inclusivity, consent documents and data collection tools will be translated and administered by trained zonal coordinators in the locally relevant languages, with verbal explanations provided in participants’ preferred language where required.

All procedures will comply with NHREC guidelines and the Declaration of Helsinki [27]. Data and samples will be de-identified, securely stored, and accessible only to authorised research personnel. Confidentiality will be maintained throughout the study and dissemination.

The principles of voluntarism, beneficence, non-maleficence, confidentiality, and veracity will be strictly upheld. Participation is entirely voluntary, with the right to withdraw at any time without affecting access to care. Risks are minimal, limited to brief discomfort during specimen collection. Potential benefits include improved understanding of the causes of neurodevelopmental conditions and access to appropriate counselling and interventions where indicated. Participation will attract no additional cost and no financial inducement; only approved transportation reimbursement will be provided. All reporting and dissemination will be truthful and transparent.

### Patient and public involvement

Patients and the public were not involved in the design of this protocol. The study is focused on a workable national screening-to-care pathway. During implementation, parents/guardians will be engaged to refine participant information materials and to guide how results and study updates are shared in a respectful, understandable format.

## Discussion

CHAMP-FX addresses a major Nigerian gap due to limited access to Fragile X molecular testing and an absence of epidemiologic data on Fragile X disorders. To our knowledge, this is the first multicentre protocol for Fragile X screening in Nigeria to implement a harmonised dried blood spot (DBS)-based workflow across the country’s six geopolitical zones. The study prioritises equity and capacity building while generating foundational data that may inform future Fragile X screening and service integration within paediatric and developmental health services. Given the 50% transmission risk from female carriers and the emergence of targeted therapeutic approaches, early identification of Fragile X syndrome may have important implications for genetic counselling, family screening and clinical management.

Operational challenges are anticipated, including DBS drying, storage and transport, laboratory turnaround time, and limited capacity for genetic counselling and follow-up. To address these challenges, the protocol incorporates standardised procedures, personnel training and defined referral pathways. The multicentre design also provides a platform for future studies, including more precise prevalence estimates and evaluation of models for integrating Fragile X screening into routine child health services.

This study has some limitations. The sample size was estimated using the best available local data, and participants will be recruited purposively from selected health facilities, which may limit the generalisability of the findings to the wider Nigerian population. Children who do not access formal health services may also be underrepresented. In addition, challenges related to counselling capacity and long-term follow-up may influence implementation across study sites. Despite these limitations, the study is expected to generate important preliminary evidence to inform future research, policy and service planning for Fragile X syndrome and premutation disorders in Nigeria.

## Conclusion

This study is the first of its kind in Nigeria and will address a long-standing gap in the recognition of Fragile X disorders. By combining molecular screening with defined pathways for genetic counselling, referral and follow-up, it is expected to generate evidence to inform the integration of Fragile X screening into paediatric and developmental health services. The protocol may also provide a framework for similar initiatives in other sub-Saharan African countries.

## Supporting information

S1 Appendix Data collection tool/proforma. (DOCX)

S2 Appendix CHAMP-FX SOP. (PDF)

S3 Appendix CHAMP-FX workflow/flowchart. (PDF)

S4 Appendix Graphical Abstract CHAMP-FX. (PPTX)

S1 Table Project Timelines. Reporting guideline: This study protocol will be reported in line with applicable reporting guidance for observational studies (STROBE) and protocol reporting requirements as required by the journal; the completed checklist will be provided as a supplementary file.
(DOCX)

## Abbreviations

BMI: Body mass index
CGG: Cytosine-guanine-guanine repeat
CHAMP-FX: Advancing Mental Health and Well-being of Nigerian Children Through Public Health Screening for Fragile X Disorders
DBS: Dried blood spot
DNA: Deoxyribonucleic acid
FMR1: Fragile X messenger ribonucleoprotein 1
FMRP: Fragile X mental retardation protein
FXAND: Fragile X–associated neuropsychiatric disorders
FXPAC: Fragile X premutation-associated conditions
FXPOI: Fragile X–associated primary ovarian insufficiency
FXS: Fragile X syndrome
FXTAS: Fragile X–associated tremor/ataxia syndrome
ID: Intellectual Disability
LMIC: Low- and middle-income country
NHREC: National Health Research Ethics Committee
PCR: Polymerase chain reaction
SDG: Sustainable Development Goals
UTR: Untranslated region
