# Supplementary material for: Advancing mental health and well-being of Nigerian children through public health screening for Fragile X disorders (CHAMP-FX): protocol of a prospective multicentre screening study with longitudinal follow-up
Source: PLoS One. 2026 Aug 5;21(8):e0355384. doi: 10.1371/journal.pone.0355384 (PMC13440841; doi:10.1371/journal.pone.0355384)
Supplement: Supplementary file 1 [file pone.0355384.s002.pdf]

CHAMP-FX STUDY STANDARD OPERATING PROCEDURE (SOP) – 31/1/2026

For Zonal Coordinators (ZCs)

Participant Recruitment, Assessment, DBS Collection and Data Handling

1. PURPOSE

To provide standardised procedures for participant recruitment, clinical assessment, dried blood spot (DBS) collection, documentation, storage, and shipment across all six geopolitical zones to ensure data and sample quality.

2. SCOPE

Applies to all Zonal Coordinators (ZCs) and Research Assistants (RAs) involved in participant enrolment and sample handling.

3. SAMPLING STRATEGY

Sampling method: Purposive sampling of eligible clinic attendees

Target per zone: 25 children (inflated from 17 for analysable samples)

Total sample: 102 participants

Population: Children 1–18 years with:

- Autism Spectrum Disorder (ASD)
- Intellectual Disability (ID)
- Global Developmental Delay (GDD)

4. A TYPICAL RESEARCH DAY WORKFLOW

STEP 1 — Identify Eligible Child

Child attending clinic with ASD, ID, or GDD.

STEP 2 — Assign Study ID

Scan QR Code from consent form

Write the QR ID on:

Hardcopy questionnaire

KoboCollect form

Whatman 903 Protein Saver Cards

⚠️ 🛑 No names on cards or lab materials

STEP 3 — Obtain Consent

Explain study in preferred language

Caregiver signs consent

Obtain child assent if ≥7 years (where feasible)

STEP 4 — Complete Data Collection

Structured interviewer-administered form (hardcopy and Kobotoolcollect):

Child data

- Age, sex
- Developmental history
- Clinical diagnosis
- Schooling status

Parent/guardian

- Age
- Education
- Occupation
- Socioeconomic indicators
- Family history

STEP 5 — Clinical Examination

Conduct and document:

- Vitals
- Anthropometry
- General physical exam
- Fragile X–associated clinical features checklist

STEP 6 — Developmental/Cognitive Testing

Age /Tool

< 4 years: ASQ-3

≥ 4 years: Raven's IQ test

Follow test manuals strictly.

Scanned informed consent form and filled out Hardcopy questionnaire should be uploaded to the Kobotoolcollect.

ZC -Keep the Hardcopy questionnaire for reference

5. DBS SAMPLE COLLECTION

Materials

Sterile lancet

Alcohol swab

Gloves

Whatman 903 Protein Saver Cards

Desiccant (silica gel preferred)

Ziploc bags

QR labels

Procedure

Wear gloves (universal precautions)

Clean finger with alcohol

Prick with sterile lancet

Allow free-flowing blood

Apply drops to fill card circles (do not layer)

Air dry horizontally for ~3 hours

Critical Handling Rules

Do NOT stack wet cards

Do NOT use heat

Do NOT touch blood spots

6. DRYING & STORAGE

After drying:

Place each card in individual Ziploc

Add desiccant

Label with QR code only

Store temporarily in freezer

⚠️ Avoid:

Contact with frost/ice

Moisture

Repeated freeze–thaw cycles

7. SHIPMENT TO CENTRAL LAB

Destination: Coordinating Molecular Lab

Nnamdi Azikiwe University, Anambra State

Batch shipment

Use approved courier

Send within 2 weeks of collection

Ensure desiccant present

8. DATA MANAGEMENT

Use QR-coded IDs only

No names on lab materials

Hard copies → Locked cabinet

Electronic data → Password-protected

Role-based access only

9. LABORATORY WORKFLOW (CENTRAL LAB)

DNA Extraction

From DBS using long-range PCR compatible protocol

PCR

Target: FMR1 CGG repeat

Long-range PCR with GC enhancers

Include:

Positive control

No-template control

Gel Check

Agarose electrophoresis

Library Prep & Sequencing

Using Oxford Nanopore Technologies ligation chemistry

Sequenced on MinION R10.4.1

Bioinformatics

Align reads to human genome

Estimate CGG repeat size

Classify allele (normal → full mutation)

10. OUTCOMES

Primary:

Proportion with FMR1 premutation/full mutation

Secondary:

CGG repeat distribution

Clinical correlations

Family cascade testing uptake

Capacity building

11. RESULTS DISCLOSURE

Confidential post-test counselling

Genetic counselling for positives

Referrals for therapy

Cascade testing offered

12. ETHICAL PRINCIPLES

NHREC approved

Voluntary participation

Right to withdraw

Minimal risk

Confidentiality maintained

13. BIAS REDUCTION

Six geopolitical zones

Standardized procedures

Central lab analysis

Pre-specified statistics

14. KEY ZC RESPONSIBILITIES

- ✔ Recruit eligible participants
- ✔ Ensure proper consent
- ✔ Accurate documentation
- ✔ Correct DBS collection

- ✔ Proper drying & storage
- ✔ Timely shipment
- ✔ Data confidentiality

15. TROUBLESHOOTING

Issue/Action

Child struggling: Pause, calm caregiver, retry safely

Insufficient blood: Use new circle

Wet storage: Discard, recollect

Damaged card: Recollect

SOP ADDENDUM — PARTICIPANT LINKAGE LOG

16. ZONAL COORDINATOR PARTICIPANT LINKAGE NOTEBOOK

To enable follow-up, result disclosure, and counselling while maintaining confidentiality:

Each Zonal Coordinator (ZC) MUST maintain a secure linkage notebook containing:

Field

Purpose

QR Study ID

Link to study records

Child's Name

For identification during follow-up

Caregiver Name

Communication

Phone Number(s)

Result disclosure & counselling

Date of Enrolment

Tracking

CONFIDENTIALITY RULES

This notebook is the ONLY document linking QR codes to participant identities.

1. It must NOT travel with samples.

2. It must NOT be photographed, digitised, or shared.
3. It must be stored in a locked cabinet.
4. Access restricted to ZC only.
5. If a ZC leaves the project, the notebook should be formally handed over to the PI.

Why this is important

Because:

Lab materials are QR-coded only

No names appear on DBS cards or databases

Follow-up, counselling, and cascade testing depend on this linkage

This ensures confidentiality + clinical responsibility.

Risk Mitigation

Risk/Control

Notebook lost: Immediate report to PI

Unauthorized access : Locked storage

Data breach: No digital copy exists

For further information, please contact PI:

Dr. Chioma N.P. Mbachu

+2348037511779

chiomambachu121417@gmail.com
