# Supplementary material for: Advancing mental health and well-being of Nigerian children through public health screening for Fragile X disorders (CHAMP-FX): protocol of a prospective multicentre screening study with longitudinal follow-up
Source: PLoS One. 2026 Aug 5;21(8):e0355384. doi: 10.1371/journal.pone.0355384 (PMC13440841; doi:10.1371/journal.pone.0355384)
Supplement: Supplementary file 2 [file pone.0355384.s003.pdf]

CHAMP-FX STUDY
ZONAL COORDINATOR WORKFLOW FLOWCHART

1. Identify Eligible Child (ASD / ID / GDD, age 1–18)

↓

2. Assign QR Study ID

↓

3. Obtain Caregiver Consent + Child Assent (if ≥7 yrs)

↓

4. Complete Questionnaire (KoboCollect + Hardcopy)

↓

5. Clinical Examination (Vitals, Anthropometry, FXS Features)

↓

6. Developmental Testing (ASQ-3 <4 yrs / Raven's ≥4 yrs)

↓

7. DBS Collection (Whatman 903 Card)

↓

8. Air Dry DBS (~3 hours)

↓

9. Package with Desiccant in Ziploc (QR Only)

↓

10. Temporary Freezer Storage (No Frost Contact)

↓

11. Batch Shipment to Central Lab (≤2 weeks)

↓

12. Laboratory Analysis

↓

13. Result Disclosure & Counselling
