# Supplementary material for: Advancing mental health and well-being of Nigerian children through public health screening for Fragile X disorders (CHAMP-FX): protocol of a prospective multicentre screening study with longitudinal follow-up
Source: PLoS One. 2026 Aug 5;21(8):e0355384. doi: 10.1371/journal.pone.0355384 (PMC13440841; doi:10.1371/journal.pone.0355384)
Supplement: Supplementary file 3 [file pone.0355384.s001.docx]

DATA COLLECTION TOOL / PROFORMA (S1 Appendix)

Questionnaire/Proforma for the CHAMP-FX Study

Advancing Mental Health and Well-Being of Nigerian Children Through Public Health Screening for Fragile X Disorders

Section A: Participant Identification

1. Study ID (check consent form for QR code): __________________________
2. Guardian’s phone number (Please call the number to ensure it works)__________________
3. Take photo of first page of consent form
4. Take photo of last page of consent form
5. Date of Enrollment: ________________

Section B: Sociodemographic Information (Circle your responses)

1. Child’s Age (in months): ________________
2. Date of Birth: ________________________
3. Gender:
   - Male
   - Female
4. State of Residence: __________________
5. Ethnic Group: _______________________
6. Geopolitical Zone: ___________________
7. Father’s Age (in years)_________
8. Mother’s age (in years)_________
9. Guardian’s age if applicable _____
10. Father’s Educational Level:
    - No Formal Education
    - Primary School
    - Secondary School
    - Tertiary Education
11. Mother’s Educational Level:
    - No Formal Education
    - Primary School
    - Secondary School
    - Tertiary Education
12. Father’s Occupation: _________
13. Mother’s Occupation: _________
14. Average monthly income_________

Section C: Medical History

1. Provisional Diagnosis (DSM 5): ________________
2. Raven Raw score Set A___________
3. Raven Raw score Set B___________
4. Raven Raw score Set C___________
5. Raven Raw score Set D___________
6. Raven Raw score Set E___________
7. Raven’s Total Raw Score__________
8. Photo of Raven’s answer sheet and upload (4 to 18 years)
9. Take a photo of the ASQ 3 form and upload for <4 years
10. Does the child have a history of developmental delays?
    - Yes
    - No
    - Unknown
11. Has the child been diagnosed with any of the following conditions? (Check all that apply):
    - Intellectual Disability a. Yes b. No
    - Autism Spectrum Disorder a. Yes b. No
    - Speech Delay a. Yes b. No
    - Motor Delay a. Yes b. No
    - Other developmental disorders (please specify): __________________
12. Family History:
    a. How many children are in the family? (siblings + index child)_____
b. Position of the index child _________
c. Is there a family history of intellectual disability, autism, or developmental delays?
   - Yes
   - No
   - Unknown

b. If yes, specify the relationship to the child (e.g., sibling, parent, etc.): __________________

13. Pregnancy and Birth History:
    a. Were there any complications during pregnancy?
    - Yes
    - No
      b. If yes, specify: _______________________

c. Mode of delivery:

- Vaginal
- Caesarean Section

d. Gestational age at birth: ___________ weeks

14. Previous Interventions/Therapies (if any):
    - Speech Therapy a. Yes b. No
    - Occupational Therapy a. Yes b. No
    - Behavioural Therapy a. Yes b. No
    - Physiotherapy a. Yes b. No
    - Others (please specify): __________________

Section D: Physical Examination Findings

1. Height (cm): ________________________
2. Weight (kg): ________________________
3. Head Circumference (cm): ____________
4. BMI________
5. Temperature (centigrade)_________
6. Pulse rate__________
7. Respiratory rate__________
8. Blood pressure
a. Systolic_____	b. diastolic______

Observed Physical Features (Tick as applicable):

| FEATURES | PRESENT | ABSENT |
| --- | --- | --- |
| Soft and velvety skin |  |  |
| Elongated face |  |  |
| Microcephaly |  |  |

FXS CLINICAL CHECKLIST 1 (tick as applicable)

Not present	Borderline or present in past	Definitely present

(score 0)	(score 1)	(score 2)

Family history of intellectual disability	[ 0 ]	[1]	[2]

Perseverative speech (repetitive speech, sounds, syllables or humming repetitively) [ 0 ]	[1]	[2]

Intellectual disability	[ 0 ]	[1]	[2]

Hand-flapping [ 0 ]	[1]	[2]

Tactilely defensive (aversion to light touch or touch normally not aversive) [ 0 ]	[1]	[2]

Hand-biting (wrist, not nails) [ 0 ]	[1]	[2]

Short attention span [ 0 ]	[1]	[2]

Hyperactivity [ 0 ]	[1]	[2]

Large testicles >2SD for age [ 0 ]	[1]	[2]

Hyperextensible Metacarpophalangeal joints (with palms flat on a table, digits 2to 5>=90^0^) [ 0 ] [1][2]

Simian crease or Sydney line[ 0 ]	[1]	[2]

Large or prominent ears [ 0 ]	[1]	[2]

Poor eye contact [ 0 ]	[1]	[2]

Total score

FRAGILE X CLINICAL CHECKLIST 2

Traits

Score 0(absent) 1(present)

Skin soft and velvety on the palms with redundancy of skin on the dorsum of hand [0]	[ 1]

Large and prominent ears [0]	[ 1 ]

Plantar crease	[0]	[ 1 ]

Familial history of Intellectual Disability [0]	[ 1 ]

Autistic-Like Behaviour	[0]	[ 1 ]

Flat feet	[0]	[ 1 ]

Large testicles	[0]	[ 1 ]

Other Notable Features or Observations: ______________

Section F: Sample Collection

1. Blood Sample Collected?
   - Yes
   - No
2. Sample ID: _________________________
3. Date of Sample Collection: __________
4. Name of Collector: _________________

Section G: Consent and Follow-Up

1. Consent Obtained:
   - Yes
   - No
2. Date of Consent: ___________________
3. Follow-Up Scheduled:
   - Yes (Date: ________________)
   - No

Definition of Terms

Intellectual disability- DSM-5 is a disorder with the onset during the developmental period that includes both intellectual deficits and adaptive functioning deficits in conceptual, social and practical domains. Please refer to DSM-5 definition of autism, global developmental delay and intellectual disability.
