# Supplementary material for: Advancing mental health and well-being of Nigerian children through public health screening for Fragile X disorders (CHAMP-FX): protocol of a prospective multicentre screening study with longitudinal follow-up
Source: PLoS One. 2026 Aug 5;21(8):e0355384. doi: 10.1371/journal.pone.0355384 (PMC13440841; doi:10.1371/journal.pone.0355384)
Supplement: Supplementary file 4 [file pone.0355384.s005.docx]

5) PROJECT TIMELINE / GANTT CHART+ KPIs (S1 Table)

| Activity/Task | Month 1-4 | Month 5-8 | Month 9-12 | Month 13-16 | Month 17-20 | Month 21-24 |
| --- | --- | --- | --- | --- | --- | --- |
| 1. Project Setup and Planning | ● |  |  |  |  |  |
| 2. Ethical Approval and Clearance | ● | ● |  |  |  |  |
| 3. Recruitment and Training | ● | ● |  |  |  |  |
| 4. Participant Enrollment |  | ● | ● |  |  |  |
| 5. Data Collection |  | ● | ● | ● |  |  |
| 6. Genetic Testing and Analysis |  |  | ● | ● |  |  |
| 7. Follow-Up and Cascade Testing |  |  |  | ● | ● |  |
| 8. Data Analysis and Interpretation |  |  |  |  | ● |  |
| 9. Dissemination of Findings |  |  |  |  |  | ● |
| 10. Evaluation and Reporting |  |  |  |  |  | ● |

| Activity/Task | Key Performance Indicator (KPI) |
| --- | --- |
| 1. Project Setup and Planning | - Project team and roles established by Month 2 |
|  | - Project management plan finalized and approved |
|  | - Monitoring and evaluation systems developed and functional |
| 2. Ethical Approval and Permissions | - Ethical approval obtained from NHREC and Ministry of Health by Month 3 |
|  | - Informed consent form finalized and ready for use |
| 3. Site Preparation and Team Training | - Staff recruitment and training completed by Month 4 |
|  | - Testing protocols and data collection systems established and tested |
| 4. Data Collection - Phase 1 | - Sociodemographic data collected from at least 85% of the participants (87/102) |
|  | - 100% physical examinations completed within the designated timeline |
|  | - 100% of blood spot samples collected and stored according to protocols |
| 5. Data Collection - Phase 2 | - Blood samples from remaining participants collected and sent for analysis by Month 9 |
|  | - At least 80% of follow-up visits conducted by Month 9 |
| 6. Data Analysis | - Initial data analysis completed by Month 10 |
|  | - Final data analysis and report draft submitted by Month 12 |
| 7. Cascade Testing and Follow-up Care | - Cascade testing conducted for at least 80% of family members of positive children |
|  | - 100% of children with positive results receive follow-up care and referrals |
| 8. Dissemination of Results | - Research report completed and submitted to funding body by Month 13 |
|  | - At least one conference presentation or publication made based on the findings by Month 14 |
|  | - Article submitted to peer-reviewed journal by Month 14 |
| 9. Project Evaluation and Closing | - Final project evaluation report completed by Month 15 |
|  | - Project documentation and close-out process finalized by Month 15 |
|  |  |
