# Supplementary material for: Advancing mental health and well-being of Nigerian children through public health screening for Fragile X disorders (CHAMP-FX): protocol of a prospective multicentre screening study with longitudinal follow-up
Source: PLoS One. 2026 Aug 5;21(8):e0355384. doi: 10.1371/journal.pone.0355384 (PMC13440841; doi:10.1371/journal.pone.0355384)
Supplement: Supplementary file 5 [file pone.0355384.s006.docx]

Thank you for the opportunity to review this interesting paper. This manuscript presents a multicentre protocol for screening Fragile X disorders among Nigerian children with neurodevelopmental conditions, addressing an important evidence gap in sub-Saharan Africa. The manuscript is generally well structured but requires **major revision** to improve clarity, writing quality, and methodological presentation before publication.

In general, the manuscript should maintain a consistent protocol-writing style. The text occasionally shifts between protocol, grant, implementation, and advocacy language, particularly in the abstract, discussion, conclusion, follow-up, and timeline sections.

**Under introduction**

Maintain consistent terminology for Fragile X conditions throughout the manuscript. Standardize the use of terms such as Fragile X syndrome, Fragile X disorders, and FMR1-related disorders. Additionally, revise the sentence beginning "Despite global advances…" for grammar, style, and capitalization, removing the unnecessary capitalized "In."

**Under objectives**

Objective number 4, which stated, (To develop a context-appropriate model for integrating Fragile X screening into paediatric/developmental health services in Nigeria.)

I have noticed that the methods do not describe how the integration model will be developed. Every study objective should correspond to a defined methodology. Therefore, I suggest to describe the methodology for developing the model (e.g., stakeholder consultation, implementation evaluation, consensus methods) or revise the objective to reflect feasibility assessment.

**Under methodology**

Study Design: the statement: “This approach enables the study to track outcomes over time, assess intervention impacts…”	The term “intervention impacts” may be misleading because this is primarily an observational screening protocol rather than an intervention trial. Therefore, replace with “assess longitudinal screening outcomes, referral processes, and implementation feasibility.”

Study Population: Children aged 1–18 years attending paediatric neurology/paediatric clinics. Briefly state how autism spectrum disorder, intellectual disability, and global developmental delay diagnoses are verified.

Sample Size:	Sample size derived from records at a single hospital. Please justify the use of patient records from one coordinating centre to estimate the sample size for a multicentre national study. Discuss any limitations of this approach and add a brief rationale explaining why local records were used and acknowledge the absence of national prevalence data.

Sampling Technique: “This pragmatic sample is intended to estimate prevalence…” Clarify that prevalence estimates apply to the selected high-risk clinical cohort rather than the general paediatric population.

Statistical Analysis: “Descriptive statistics will be done…” Use standard statistical terminology throughout.	Replace “will be done” with “will be performed” and “frequencies/percentages will be calculated.”

Statistical Analysis: “Logistic regression may be explored…”	Clarify under what circumstances regression modelling will be performed (e.g., sufficient number of positive cases).

**Result disclosure, counselling, and follow-up**

In the manuscript, it is mentioned that targeted therapy will be offered using metformin for participants diagnosed with FXS. As written, this section could be interpreted as moving from screening into therapeutic intervention without adequate explanation. The wording should be revised carefully to avoid ambiguity and to maintain a protocol-appropriate scope. Therefore, I suggest to clearly distinguishing:

- what is part of the research protocol,
- what is part of post-test counselling,
- what constitutes standard clinical referral,
- and what, if any, treatment is outside the study but may be discussed or offered in routine care.

**Under discussion**

The discussion should focus on the anticipated contribution of the protocol rather than expected outcomes. Since this is a protocol paper, statements regarding improved clinical outcomes, national impact, or service transformation should be presented as potential benefits rather than established or expected results.

This is where the manuscript shifts most from a protocol to advocacy.

Examples include statements such as:

- the study **"will substantially improve care"**
- the project **"will transform diagnosis and management"**
- the programme **"will improve the mental health and well-being of Nigerian children"**
- statements implying that the screening programme **will be successfully scaled nationally**.

It is important to keep in mind that the discussion should acknowledge limitations more directly. These likely include:

- relatively small sample size,
- purposive rather than population-based recruitment,
- recruitment from selected health facilities,
- limited generalisability to all Nigerian children,
- possible underrepresentation of children outside formal care pathways,
- and feasibility challenges for counselling and follow-up.

**Under Conclusion**

The Conclusion reads more like the conclusion of a completed study than a study protocol. Rewrite with more focus on emphasize the anticipated contribution of the proposed study rather than implying demonstrated effectiveness or future implementation success.

**Under Appendix**

Ensure all appendices and supplementary materials are cited at their first mention in the manuscript. Several supporting documents (e.g., data collection proforma, examination checklists, workflow flowchart, SOP, and project timeline) are not consistently referenced.For example, CHAMP-FX Workflow Flowchart, It is listed at the end of the PDF but not cited in the manuscript. Please cite it in the Laboratory Workflow section

(S1 Appendix)

**The statement : “Fragile X syndrome is a genetic condition caused by changes in the FMR1 gene on the X chromosome.”** This is accurate, but may be difficult for some participants. Consider a plainer explanation such as **“Fragile X syndrome is an inherited condition caused by a change in a gene.”**

The word **“premutation”** may not be understandable to non-specialists. It should either be explained in plain language or omitted from the consent form if not essential for participant understanding.
