# Supplementary material for: Advancing mental health and well-being of Nigerian children through public health screening for Fragile X disorders (CHAMP-FX): protocol of a prospective multicentre screening study with longitudinal follow-up
Source: PLoS One. 2026 Aug 5;21(8):e0355384. doi: 10.1371/journal.pone.0355384 (PMC13440841; doi:10.1371/journal.pone.0355384)
Supplement: Supplementary file 6 [file pone.0355384.s008.docx]

**Response to Reviewer**

We sincerely thank the editor and reviewers for the thorough review of our work and the constructive feedback, which has significantly strengthened our manuscript. We have carefully revised the manuscript to improve clarity, methodological rigour, consistency of terminology, and adherence to a protocol-writing style. Our detailed responses are provided below.

Editor

1.**Editor comment:** Please ensure that your manuscript meets PLOS ONE's style requirements, including those for file naming. The PLOS ONE style templates can be found at

<https://journals.plos.org/plosone/s/file?id=wjVg/PLOSOne_formatting_sample_main_body.pdf> and

[https://journals.plos.org/plosone/s/file?id=ba62/PLOSOne_formatting_sample_title_authors_affiliations.pdf](https://track.editorialmanager.com/CL0/https:%2F%2Fjournals.plos.org%2Fplosone%2Fs%2Ffile%3Fid=ba62%2FPLOSOne_formatting_sample_title_authors_affiliations.pdf/1/010f019f2bb17df8-b232ca4f-5e08-4ef4-9057-6efc0a8e03bd-000000/u513CCo3M6uPdxRQQyjfCKWszXMQHTZmQsslVSU748c=258)

**Response:** The corrections have been implemented throughout the manuscript in line with PLOS One guidelines.

2. **Editor comment:** Thank you for stating the following financial disclosure:

“This work was supported by the TETFund National Research Fund (NRF) [Grant No. TETFund/ES/DR&D-CE/NRF2024/SETI/HSW/00032/VOL.1]”

Please state what role the funders took in the study.  If the funders had no role, please state: "The funders had no role in study design, data collection and analysis, decision to publish, or preparation of the manuscript."

**Response:** The statement has been added to the cover letter in line with the comment - "The funders had no role in study design, data collection and analysis, decision to publish, or preparation of the manuscript."

3. **Editor comment:** *Thank you for stating the following in the Competing Interests section:*

*“Randi Hagerman (RH) reports research grants from Zynerba/Harmony for a cannabidiol (CBD) trial in Fragile X syndrome, and from Shionogi & Co., Ltd. for a zatolmilast study, awarded to UC Davis Medical Center.*

*RH also serves on advisory boards for Shionogi and Harmony Biosciences and is a member of the Clinical Trials Committee of the National Fragile X Foundation.*

*These relationships are unrelated to the current study and did not influence the study design, data collection, analysis, interpretation, or decision to publish.*

*All other authors declare no competing interests.”*

*We note that one or more of the authors are employed by a commercial company: “Zynerba/Harmony, Shionogi and Harmony Biosciences”.*

*1. Please provide an amended Funding Statement declaring this commercial affiliation, as well as a statement regarding the Role of Funders in your study. If the funding organization did not play a role in the study design, data collection and analysis, decision to publish, or preparation of the manuscript and only provided financial support in the form of authors' salaries and/or research materials, please review your statements relating to the author contributions, and ensure you have specifically and accurately indicated the role(s) that these authors had in your study. You can update author roles in the Author Contributions section of the online submission form.*

*Please also include the following statement within your amended Funding Statement.*

*“The funder provided support in the form of salaries for authors “R.H.” but did not have any additional role in the study design, data collection and analysis, decision to publish, or preparation of the manuscript. The specific roles of these authors are articulated in the ‘author contributions’ section.”*

*If your commercial affiliation did play a role in your study, please state and explain this role within your updated Funding Statement.*

**Response:** Thank you for your comment. We wish to clarify that Prof. Randi Hagerman is not employed by Zynerba, Harmony Biosciences or Shionogi. She is employed by the University of California, Davis, USA. The competing interests statement refers to research grants awarded to UC Davis Medical Center for unrelated clinical trials and advisory board memberships. No commercial company funded the present study or provided salary support for any author. Accordingly, the requested statement regarding salary support from a commercial funder is not applicable. We clarify that the commercial entities had no role in the study design, data collection, analysis, interpretation, manuscript preparation or decision to publish.

3. **Editor comment:** *Please also provide an updated Competing Interests Statement declaring this commercial affiliation along with any other relevant declarations relating to employment, consultancy, patents, products in development, or marketed products, etc.*

*Within your Competing Interests Statement, please confirm that this commercial affiliation does not alter your adherence to all PLOS ONE policies on sharing data and materials by including the following statement: "This does not alter our adherence to  PLOS ONE policies on sharing data and materials.” (as detailed online in our guide for authors* [*http://journals.plos.org/plosone/s/competing-interests*](http://journals.plos.org/plosone/s/competing-interests)*) . If this adherence statement is not accurate and  there are restrictions on sharing of data and/or materials, please state these. Please note that we cannot proceed with consideration of your article until this information has been declared.*

*Please include both an updated Funding Statement and Competing Interests Statement in your cover letter. We will change the online submission form on your behalf.*

**Response:** Updated Funding Statement

Funding: This study was funded by the Tertiary Education Trust Fund (TETFund), National Research Fund (Grant No. TETFund/ES/DR&D-CE/NRF2024/SETI/HSW/00032/VOL.1). Prof. Randi Hagerman's unrelated institutional research at the University of California, Davis has received research funding from Zynerba/Harmony and Shionogi for Fragile X clinical trials. These commercial entities did not provide funding for the present study and had no role in the study design, data collection, data analysis, interpretation of the data, decision to publish, or preparation of the manuscript.

Updated Competing Interests Statement

Competing interests: Prof. Randi Hagerman reports unrelated institutional research grants awarded to the University of California, Davis from Zynerba/Harmony and Shionogi for Fragile X clinical trials. She also serves on advisory boards for Harmony Biosciences and Shionogi and is a member of the Clinical Trials Committee of the National Fragile X Foundation. These relationships are unrelated to the present study and did not influence the study design, data collection, analysis, interpretation, or decision to publish. All other authors declare no competing interests. This does not alter our adherence to PLOS ONE policies on sharing data and materials.

**4.  Editor comment:** *When completing the data availability statement of the submission form, you indicated that you will make your data available on acceptance. We strongly recommend all authors decide on a data sharing plan before acceptance, as the process can be lengthy and hold up publication timelines. Please note that, though access restrictions are acceptable now, your entire data will need to be made freely accessible if your manuscript is accepted for publication. This policy applies to all data except where public deposition would breach compliance with the protocol approved by your research ethics board. If you are unable to adhere to our open data policy, please kindly revise your statement to explain your reasoning and we will seek the editor's input on an exemption. Please be assured that, once you have provided your new statement, the assessment of your exemption will not hold up the peer review process.*

**Response:** The Data Availability Statement has been modified to: No datasets were generated or analysed during the preparation of this study protocol. Following completion of the study, the de-identified dataset underlying the findings will be deposited in an appropriate publicly accessible data repository prior to publication, in accordance with the PLOS ONE Data Availability Policy, applicable ethical approvals, and participant confidentiality requirements.

5. **Editor comment:** *Your ethics statement should only appear in the Methods section of your manuscript. If your ethics statement is written in any section besides the Methods, please move it to the Methods section and delete it from any other section. Please ensure that your ethics statement is included in your manuscript, as the ethics statement entered into the online submission form will not be published alongside your manuscript.*

**Response:**  The ethics statement has been removed from the abstract section of the manuscript.

**6. Editor comment:** *If the reviewer comments include a recommendation to cite specific previously published works, please review and evaluate these publications to determine whether they are relevant and should be cited. There is no requirement to cite these works unless the editor has indicated otherwise.*

**Response:**  Thank you. The reviewer's comments do not include such a recommendation.

**General comment**

**Reviewer comment:** *The manuscript occasionally shifts between protocol, grant, implementation and advocacy language.*

**Response:** Thank you for this important observation. We have carefully revised the manuscript throughout to maintain a consistent protocol-writing style. Statements implying demonstrated effectiveness, service transformation or implementation success have been moderated to reflect the anticipated contribution of the proposed study. These revisions were made throughout the Abstract, Discussion, Conclusion and other relevant sections.

**Introduction**

**Reviewer comment:** *Maintain consistent terminology for Fragile X conditions throughout the manuscript. Revise the sentence beginning "Despite global advances…"*

**Response:** Thank you for this suggestion. We have standardised the terminology used throughout the manuscript to ensure consistency in the description of Fragile X disorders. The introductory section has also been revised for grammar, clarity and style, including the sentence beginning "Despite global advances…". These changes are reflected on **Page 4, Lines 95–117**.

**Objectives**

**Reviewer comment:** *Objective 4 should correspond to a defined methodology.*

**Response:** We appreciate this observation. We retained Objective 4 and added a corresponding methodology describing how the integration model will be developed. Specifically, the revised manuscript now describes stakeholder engagement involving the Child Neurology Society of Nigeria, Paediatric Association of Nigeria, the Federal Ministry of Health, selected State Ministries of Health, the National Primary Health Care Development Agency, and other relevant stakeholders. Findings from the study together with stakeholder recommendations will be synthesised to develop a context-appropriate model for integrating Fragile X screening into paediatric and developmental health services in Nigeria. These revisions are presented on **Page 12, Lines 301–310**.

**Methodology**

**Study design**

**Reviewer comment:** *Replace "assess intervention impacts".*

**Response:** Thank you. The wording has been revised to more accurately reflect the observational nature of the study. The manuscript now states that the study will assess longitudinal screening outcomes, referral processes and implementation outcomes rather than intervention impacts. These changes are reflected on **Page 6, Lines 143–149**.

**Study population**

**Reviewer comment:** *Briefly state how autism spectrum disorder, intellectual disability and global developmental delay diagnoses are verified.*

**Response:** We have expanded this section to describe the diagnostic procedures. Autism spectrum disorder, intellectual disability and global developmental delay are confirmed using DSM-5 diagnostic criteria through multidisciplinary assessments conducted by trained neurodevelopmental paediatricians, paediatric neurologists and a psychologist. We also describe the use of the Ages and Stages Questionnaire (ASQ-3) for children younger than four years and the Raven's Standard Progressive Matrices (Revised 2019) for children aged four years and above. These revisions appear on **Pages 6–7, Lines 156–168**.

**Sample size**

**Reviewer comment:** *Justify the use of patient records from one coordinating centre.*

**Response:** Thank you for this valuable comment. We have added a rationale explaining that, in the absence of national prevalence data on Fragile X syndrome among high-risk Nigerian children, patient records from the coordinating centre represented the best available local data for estimating the sample size. We also acknowledge the limitation of using a single-centre estimate while noting that the multicentre design enhances the representativeness of the study. These revisions are presented on **Pages 7–8, Lines 181–194**.

**Sampling technique**

**Reviewer comment:** *Clarify that prevalence estimates apply to the selected high-risk cohort.*

**Response:** The manuscript has been revised accordingly. It now clearly states that prevalence estimates apply to the selected high-risk clinical cohort rather than the general paediatric population. This clarification appears on **Page 8, Lines 195–200**.

**Statistical analysis**

**Reviewer comment:** *Use standard statistical terminology.*

**Response:** We have revised the statistical analysis section using standard statistical terminology. Phrases such as "will be done" have been replaced with "will be performed", and "frequencies and percentages will be calculated". These revisions are found on **Pages 10–11, Lines 262–267**.

**Reviewer comment:** *Clarify under what circumstances logistic regression modelling will be performed.*

**Response:** We have clarified that logistic regression analyses will only be performed if sufficient Fragile X-positive cases are available to support reliable multivariable modelling. Where the number of positive cases is insufficient, analyses will be limited to descriptive and bivariate methods. These revisions are presented on **Page 11, Lines 268–272**.

**Result disclosure, counselling and follow-up**

**Reviewer comment:** *Clearly distinguish research procedures, post-test counselling, routine clinical referral and treatment outside the study.*

**Response:** We appreciate this important observation. This section has been substantially revised to clearly distinguish activities that form part of the research protocol from routine clinical care. The revised manuscript now specifies that the research protocol includes result disclosure through post-test genetic counselling, while routine clinical management consists of referral to trained paediatric neurologists at participating centres, with referral to the coordinating centre where specialist input is required. Any treatment, including consideration of metformin where clinically indicated, will be provided as part of routine clinical care and is outside the scope of the research protocol. These revisions are presented on **Pages 11–12, Lines 278–310**.

**Discussion**

**Reviewer comment:** *Focus the discussion on the anticipated contribution of the protocol and acknowledge study limitations.*

**Response:** Thank you for this insightful suggestion. The Discussion has been extensively revised to maintain a protocol-focused perspective. Statements implying demonstrated effectiveness or future implementation success have been replaced with more appropriate language describing the anticipated contribution of the study. We have also expanded the limitations section to acknowledge the relatively small sample size, purposive sampling, recruitment from selected health facilities, limited generalisability, possible underrepresentation of children outside formal healthcare settings, and potential challenges related to counselling and long-term follow-up. These revisions are presented on **Pages 13–14, Lines 337–357**.

**Conclusion**

**Reviewer comment:** *Rewrite the Conclusion to emphasise the anticipated contribution of the proposed study.*

**Response:** The Conclusion has been revised to better reflect the purpose of a study protocol. It now emphasises the anticipated contribution of CHAMP-FX without implying demonstrated effectiveness or implementation success. These revisions are presented on **Page 15, Lines 371–380**.

**Appendices**

**Reviewer comment:** *Ensure all appendices and supplementary materials are cited appropriately.*

**Response:** We have reviewed the manuscript and ensured that all appendices and supplementary materials are cited at their first mention, including the informed consent form, data collection tool, workflow and project timeline. Relevant citations have been added throughout the Methods section.

**Consent form**

**Reviewer comment:** *Simplify the explanation of Fragile X syndrome and explain the term "premutation".*

**Response:** Thank you for this helpful suggestion. The participant information sheet has been revised using simpler language appropriate for non-specialist participants. We also replaced technical terminology where possible and included a brief plain-language explanation of the term "premutation" to improve participant understanding. These revisions are presented on **Page 23, Lines 552–564**.

Once again, thank you for the critical review of the manuscript.

Response to Reviewer 2

We sincerely thank the reviewer for the careful and constructive review of our manuscript. We appreciate the insightful comments and recommendations, which have significantly improved the clarity, methodological rigour and overall quality of the protocol. Our responses are provided below.

Major comments

*Comment: Please include the word "Protocol" in your title as per PLOS ONE recommendations.*

Response: Thank you for this helpful suggestion. We have revised the title to include the word "Protocol" in accordance with the PLOS ONE recommendations. These changes are reflected on **Page 1, Lines 1–2**.

Comment: *Please ensure the sample-size formula is displayed correctly and replace "will be calculated" with "was calculated".*

Response: Thank you. The formula has been reformatted to display correctly and the text has been revised to indicate that the sample size was calculated, reflecting the completed study design. These changes are reflected on **Page 7, Lines 181–188**.

Comment: *Please elaborate on the validated DNA extraction workflow.*

Response: Thank you for this valuable suggestion. We have expanded the laboratory workflow to provide additional methodological detail, including the DNA extraction protocol, quality assessment procedures, long-range PCR workflow and sequencing methodology to improve reproducibility. These changes are reflected on **Page 9, Lines 217–228**.

Comment: *Please mention in the Methods that findings will be reported according to STROBE.*

Response: Thank you. We have added a statement to the Methods indicating that the study findings will be reported in accordance with the Strengthening the Reporting of Observational Studies in Epidemiology (STROBE) Statement. These changes are reflected on **Page 11, Lines 268–270**

Comment: *The phrase "where feasible" is too weak for transfer of identifiable or genetic data.*

Response: We agree. The wording has been revised to specify the minimum security measures that will be employed for storage and transfer of identifiable and genetic data, including password-protected databases, encrypted electronic data transfer and restricted role-based access. These changes are reflected on **Page 11, Lines 275–278**

Comment: *The discussion states that the study will generate "robust data". Given the sample size and purposive sampling, "foundational" or "pilot" data would be more appropriate.*

Response: Thank you for this important observation. We have revised the wording to describe the anticipated outputs as foundational evidence rather than robust data. CHAMP-FX is a nationally funded multicentre screening study rather than a pilot or feasibility study; therefore, we have retained this description while clarifying that the findings will provide preliminary evidence from a purposively selected high-risk clinical cohort. These changes are reflected on **Page 14, Line 355**

Comment: *The sample-size calculation is a major weakness.*

Response: We appreciate the reviewer's thoughtful comments regarding the sample-size calculation. In the absence of national prevalence data on Fragile X syndrome among high-risk Nigerian children, the sample size was estimated using the best available local data from the coordinating centre. We have clarified that the prevalence estimates relate to the selected high-risk clinical cohort rather than the general paediatric population and have acknowledged the limitations associated with this approach. We have also clarified that logistic regression analyses will only be undertaken if sufficient Fragile X-positive cases are available to support reliable multivariable modelling. While recognising the limitations, we believe this nationally funded multicentre study will provide important foundational evidence on Fragile X disorders among high-risk Nigerian children and inform future larger studies. These changes are reflected on **Page 15, Lines 366 - 373**

Minor comments

Comment: *"In" should not be capitalised.*

Response: Corrected. These changes are reflected on **Page 4, Line 116**

Comment: *Expand CHAMP-FX upon first use.*

Response: Revised accordingly. These changes are reflected on **Page 3, Lines 74 - 75**

Comment: *Move the Objectives section to the Methods.*

Response: Thank you for the suggestion. We have retained the Objectives immediately following the Introduction to improve readability and maintain consistency with the journal's protocol format.

Comment: *Change the final sentence to "See Supplementary Material S1..."*

Response: Revised as suggested. These changes are reflected on **Page 3, Lines 74 - 75**

Comment: *Please be consistent with capitalisation.*

Response: Revised throughout.

Comment: *Line 193 contains language issues.*

Response: The sentence has been revised for clarity and grammar. These changes are reflected on **Page 8**

Comment: *Specify the refrigeration temperature.*

Response: The storage temperature has now been specified. These changes are reflected on **Page 9, Line 213**

Comment: *Clarify what is meant by courier service.*

Response: We have clarified that samples will be transported using a dedicated medical courier service that complies with applicable biospecimen transport requirements. These changes are reflected on **Page 9, Line 214 - 216**

Comment: *Use the abbreviation NTC.*

Response: Revised accordingly. These changes are reflected on **Page 10, Line 242**

Comment: *Expand UCD.*

Response: Revised to University of California, Davis (UCD) at first mention. These changes are reflected on **Page 10, Lines 228- 229**

Comment: *Insert a full stop before "PCR..."*

Response: Sentence was rephrased.

Comment: *Expand abbreviation at first use.*

Response: Revised accordingly.

Comment: *Replace the semicolon with a full stop.*

Response: Corrected.

Comment: *Insert a missing full stop.*

Response: Corrected.

Comment: *Replace the Stata citation.*

Response: Revised to Stata (Version 16.0; StataCorp LLC, College Station, TX, USA). This change is reflected on **Page 11, Line 283**

Comment: *Please cite the Declaration of Helsinki.*

Response: The appropriate citation has been added. This change is reflected on **Page 13, Line 337**

Comment: *The Consent for Publication declaration is unnecessary.*

Response: Removed. This change is reflected on **Page 14, Lines 511 -513**

Comment: *DSM-5 and other diagnostic criteria should also be mentioned in the main manuscript.*

Response: We agree. Additional methodological details describing the diagnostic criteria have been incorporated into the Study Population section. This change is reflected on **Page 7, Lines 159 - 167**

Comment: *Please provide more detail regarding the laboratory workflow.*

Response: The laboratory workflow has been expanded to improve reproducibility and provide greater methodological detail, including DNA extraction, quality assessment, long-range PCR, sequencing procedures and quality control. This change is reflected on **Page 10, Lines 219 -234**

Comment: *Consider producing a graphical abstract.*

Response: We thank the reviewer for this valuable suggestion. A graphical abstract illustrating the overall study workflow has been developed and included to enhance the presentation of the protocol.

We sincerely thank the reviewer for these thoughtful comments. The revisions have substantially strengthened the manuscript by improving methodological clarity, transparency and consistency with PLOS ONE guidance.
