# Supplementary material for: Advancing mental health and well-being of Nigerian children through public health screening for Fragile X disorders (CHAMP-FX): protocol of a prospective multicentre screening study with longitudinal follow-up
Source: PLoS One. 2026 Aug 5;21(8):e0355384. doi: 10.1371/journal.pone.0355384 (PMC13440841; doi:10.1371/journal.pone.0355384)
Supplement: Peer review history

# Author comment: Author response to Decision Letter 0

**DOI:** 10.1371/journal.pone.0355384.r001

**Transfer Alert**

This paper was transferred from another journal. As a result, its full editorial history (including decision letters, peer reviews and author responses) may not be present.

13 May 2026

# Peer review: Decision Letter 0

**DOI:** 10.1371/journal.pone.0355384.r002

**Authors:** David Ikwuka

4 Jul 2026

PONE-D-26-20888

Advancing mental health and well-being of Nigerian children through public health screening for Fragile X disorders (CHAMP-FX): a prospective multicentre screening study with longitudinal follow-up

PLOS One

Dear Dr. Mbachu,

Thank you for submitting your manuscript to PLOS ONE. After careful consideration, we feel that it has merit but does not fully meet PLOS ONE’s publication criteria as it currently stands. Therefore, we invite you to submit a revised version of the manuscript that addresses the points raised during the review process.

Please submit your revised manuscript by Aug 03 2026 11:59PM. If you will need more time than this to complete your revisions, please reply to this message or contact the journal office at plosone@plos.org. When you're ready to submit your revision, log on to https://www.editorialmanager.com/pone/ and select the 'Submissions Needing Revision' folder to locate your manuscript file.

Please include the following items when submitting your revised manuscript:

- A letter that responds to each point raised by the academic editor and reviewer(s). You should upload this letter as a separate file labeled 'Response to Reviewers'.
- A marked-up copy of your manuscript that highlights changes made to the original version. You should upload this as a separate file labeled 'Revised Manuscript with Track Changes'.
- An unmarked version of your revised paper without tracked changes. You should upload this as a separate file labeled 'Manuscript'.

If you would like to make changes to your financial disclosure, please include your updated statement in your cover letter. Guidelines for resubmitting your figure files are available below the reviewer comments at the end of this letter.

If applicable, we recommend that you deposit your laboratory protocols in protocols.io to enhance the reproducibility of your results. Protocols.io assigns your protocol its own identifier (DOI) so that it can be cited independently in the future. For instructions see: https://journals.plos.org/plosone/s/submission-guidelines#loc-laboratory-protocols. Additionally, PLOS ONE offers an option for publishing peer-reviewed Lab Protocol articles, which describe protocols hosted on protocols.io. Read more information on sharing protocols at https://plos.org/protocols?utm_medium=editorial-email&utm_source=authorletters&utm_campaign=protocols.

As the corresponding author, your ORCID iD is verified in the submission system and will appear in the published article. PLOS supports the use of ORCID, and we encourage all coauthors to register for an ORCID iD and use it as well. Please encourage your coauthors to verify their ORCID iD within the submission system before final acceptance, as unverified ORCID iDs will not appear in the published article. *Only*  the individual author can complete the verification step; PLOS staff *cannot*  verify ORCID iDs on behalf of authors.

We look forward to receiving your revised manuscript.

Kind regards,

David Chibuike Ikwuka, Ph.D.

Academic Editor

PLOS One

Journal Requirements:

When submitting your revision, we need you to address these additional requirements.

1.Please ensure that your manuscript meets PLOS ONE's style requirements, including those for file naming. The PLOS ONE style templates can be found at

https://journals.plos.org/plosone/s/file?id=wjVg/PLOSOne_formatting_sample_main_body.pdf and

https://journals.plos.org/plosone/s/file?id=ba62/PLOSOne_formatting_sample_title_authors_affiliations.pdf

2. Thank you for stating the following financial disclosure:

“This work was supported by the TETFund National Research Fund (NRF) [Grant No. TETFund/ES/DR&D-CE/NRF2024/SETI/HSW/00032/VOL.1]”

Please state what role the funders took in the study.  If the funders had no role, please state: "The funders had no role in study design, data collection and analysis, decision to publish, or preparation of the manuscript."

If this statement is not correct you must amend it as needed.

Please include this amended Role of Funder statement in your cover letter; we will change the online submission form on your behalf.

3. Thank you for stating the following in the Competing Interests section:

“Randi Hagerman (RH) reports research grants from Zynerba/Harmony for a cannabidiol (CBD) trial in Fragile X syndrome, and from Shionogi & Co., Ltd. for a zatolmilast study, awarded to UC Davis Medical Center.

RH also serves on advisory boards for Shionogi and Harmony Biosciences and is a member of the Clinical Trials Committee of the National Fragile X Foundation.

These relationships are unrelated to the current study and did not influence the study design, data collection, analysis, interpretation, or decision to publish.

All other authors declare no competing interests.”

We note that one or more of the authors are employed by a commercial company: “Zynerba/Harmony, Shionogi and Harmony Biosciences”.

1. Please provide an amended Funding Statement declaring this commercial affiliation, as well as a statement regarding the Role of Funders in your study. If the funding organization did not play a role in the study design, data collection and analysis, decision to publish, or preparation of the manuscript and only provided financial support in the form of authors' salaries and/or research materials, please review your statements relating to the author contributions, and ensure you have specifically and accurately indicated the role(s) that these authors had in your study. You can update author roles in the Author Contributions section of the online submission form.

Please also include the following statement within your amended Funding Statement.

“The funder provided support in the form of salaries for authors “R.H.” but did not have any additional role in the study design, data collection and analysis, decision to publish, or preparation of the manuscript. The specific roles of these authors are articulated in the ‘author contributions’ section.”

If your commercial affiliation did play a role in your study, please state and explain this role within your updated Funding Statement.

2. Please also provide an updated Competing Interests Statement declaring this commercial affiliation along with any other relevant declarations relating to employment, consultancy, patents, products in development, or marketed products, etc.

Within your Competing Interests Statement, please confirm that this commercial affiliation does not alter your adherence to all PLOS ONE policies on sharing data and materials by including the following statement: "This does not alter our adherence to  PLOS ONE policies on sharing data and materials.” (as detailed online in our guide for authors http://journals.plos.org/plosone/s/competing-interests) . If this adherence statement is not accurate and  there are restrictions on sharing of data and/or materials, please state these. Please note that we cannot proceed with consideration of your article until this information has been declared.

Please include both an updated Funding Statement and Competing Interests Statement in your cover letter. We will change the online submission form on your behalf.

4. When completing the data availability statement of the submission form, you indicated that you will make your data available on acceptance. We strongly recommend all authors decide on a data sharing plan before acceptance, as the process can be lengthy and hold up publication timelines. Please note that, though access restrictions are acceptable now, your entire data will need to be made freely accessible if your manuscript is accepted for publication. This policy applies to all data except where public deposition would breach compliance with the protocol approved by your research ethics board. If you are unable to adhere to our open data policy, please kindly revise your statement to explain your reasoning and we will seek the editor's input on an exemption. Please be assured that, once you have provided your new statement, the assessment of your exemption will not hold up the peer review process.

5. Your ethics statement should only appear in the Methods section of your manuscript. If your ethics statement is written in any section besides the Methods, please move it to the Methods section and delete it from any other section. Please ensure that your ethics statement is included in your manuscript, as the ethics statement entered into the online submission form will not be published alongside your manuscript.

6. If the reviewer comments include a recommendation to cite specific previously published works, please review and evaluate these publications to determine whether they are relevant and should be cited. There is no requirement to cite these works unless the editor has indicated otherwise.

[Note: HTML markup is below. Please do not edit.]

Reviewer's Responses to Questions

**Comments to the Author**

1. Does the manuscript provide a valid rationale for the proposed study, with clearly identified and justified research questions?

The research question outlined is expected to address a valid academic problem or topic and contribute to the base of knowledge in the field.

Reviewer #1: Yes

Reviewer #2: Yes

**********

2. Is the protocol technically sound and planned in a manner that will lead to a meaningful outcome and allow testing the stated hypotheses?

The manuscript should describe the methods in sufficient detail to prevent undisclosed flexibility in the experimental procedure or analysis pipeline, including sufficient outcome-neutral conditions (e.g. necessary controls, absence of floor or ceiling effects) to test the proposed hypotheses and a statistical power analysis where applicable. As there may be aspects of the methodology and analysis which can only be refined once the work is undertaken, authors should outline potential assumptions and explicitly describe what aspects of the proposed analyses, if any, are exploratory.

Reviewer #1: Yes

Reviewer #2: Partly

**********

3. Is the methodology feasible and described in sufficient detail to allow the work to be replicable?

Descriptions of methods and materials in the protocol should be reported in sufficient detail for another researcher to reproduce all experiments and analyses. The protocol should describe the appropriate controls, sample size calculations, and replication needed to ensure that the data are robust and reproducible.

Reviewer #1: Yes

Reviewer #2: Yes

**********

4. Have the authors described where all data underlying the findings will be made available when the study is complete?

The PLOS Data policy requires authors to make all data underlying the findings described in their manuscript fully available without restriction, with rare exception, at the time of publication. The data should be provided as part of the manuscript or its supporting information, or deposited to a public repository. For example, in addition to summary statistics, the data points behind means, medians and variance measures should be available. If there are restrictions on publicly sharing data—e.g. participant privacy or use of data from a third party—those must be specified.

Reviewer #1: Yes

Reviewer #2: No

**********

5. Is the manuscript presented in an intelligible fashion and written in standard English?

PLOS ONE does not copyedit accepted manuscripts, so the language in submitted articles must be clear, correct, and unambiguous. Any typographical or grammatical errors should be corrected at revision, so please note any specific errors here.

Reviewer #1: Yes

Reviewer #2: Yes

**********

6. Review Comments to the Author

Please use the space provided to explain your answers to the questions above and, if applicable, provide comments about issues authors must address before this protocol can be accepted for publication. You may also include additional comments for the author, including concerns about research or publication ethics.

You may also provide optional suggestions and comments to authors that they might find helpful in planning their study.

(Please upload your review as an attachment if it exceeds 20,000 characters)

Reviewer #1: Reviewer comments

06/06/2026

ID: PONE-D-26-20888

Title: Advancing mental health and well-being of Nigerian children through public health screening for Fragile X disorders (CHAMP-FX): a prospective multicentre screening study with longitudinal follow-up

Dear Editor and Authors,

Thank you for giving me the opportunity to read the present work and evaluate the study. I found the protocol quite interesting to read regarding this prospective study in Nigeria advancing pediatric neurodegenerative disease evaluation in the African continent. I believe the present work is quite strong, rigorous, and provides a strong foundation for this multi centred study around Nigeria. In this ongoing study I wish the present authors the best of luck.

This manuscript addresses an important gap in Fragile X diagnosis and public-health genetics in Nigeria and has several strengths, including a multicentre design, planned DBS-based molecular screening, and integration of counselling/referral pathways. The methods used in the study are also described in sufficient detail and quite rigorously which aligns with PLOS One’s goals of reproducible and transparent research.

Despite this I have a few comments that I believe the author should address to make this work a publishable protocol. Kindly find my comments below.

Major Comments

- Please include the word “Protocol” in your title as per PLOS One recommendations.

-Line 170, please ensure the formula is displayed correctly, as it seems to have lost correct display. I would also recommend saying that the sample size “was” calculated instead of “will” be calculated.

- Line 201 please elaborate what the validated workflow is. This section (200 DNA extraction and quality assessment) needs to be more thorough for a protocol.

-The use of STROBE is mentioned in the appendix. Please ensure that you also mention that findings will be reported as per STROBE in the methods section.

- The phrase “where feasible” is too weak for transfer of identifiable or genetic data. The protocol should state the actual minimum required security practices.

- The discussion says the study will generate “robust data,” but given purposive sampling and n = 102, “foundational” or “pilot” data would be more accurate.

- The sample-size calculation is a major weakness. The authors use P = 0.023, then apply a finite population correction using records from a single centre: 12 autism cases every 5 months at NAUTH, estimated as 28.9 per year. This produces an adjusted minimum sample size of 15.7, after which the study selects 17 participants per zone, totalling 102. The value P = 0.023 appears to relate to autism prevalence rather than the expected prevalence of FMR1 expansion among the study’s target population. If the primary outcome is Fragile X full mutation/premutation positivity, the sample-size calculation should be based on the expected FMR1 positivity rate or on desired precision around that estimate. The finite population correction is not appropriate as presented. The denominator is based only on NAUTH autism clinic records, not on the six-site target population and not on all included diagnoses, namely ID, ASD, and GDD. With n = 102, if the expected FMR1 expansion rate is low, the number of positive cases may be very small. The planned association testing and possible logistic regression may therefore be infeasible. The statistical plan says logistic regression “may be explored for correlates of screen positivity if event counts permit,” but this needs a clearer decision rule and a more cautious framing. The authors should provide either a precision-based calculation for prevalence/proportion estimation (which would lead to an inclusion population much higher than 102) or explicitly describe this as a feasibility/pilot screening study rather than a robust prevalence study. The present proposal, regardless, will provide an idea regarding the prevalence of Fragile X in Nigeria.

Minor Comments

-Line 115 “In” should not be capitalized.

-Line 123 please expand CHAMP-FX upon first use, even if the expansion is there in the abstract.

- I recommend moving the “objectives” section to be part of the material and methods (specifically moving the main and specific objectives bullets) while adding a more narrative and small paragraph at the end of the introduction to briefly explore the objectives. This is just for ease of readership and to highlight the specific objectives as part of the methods.

-Line 168 please consider changing the last sentence to: “See Supplementary Material S1 for the Informed Consent Form.”

- Line 185 and 186 please be consistent with capitalization.

- Line 193 seems to have language structural issues. Please fix.

- Line 196 it would be a good idea to specify the temperature of the refrigeration.

- Line 197 please specify what you mean by courier service in the context of Nigeria. Do you mean it will be shipped by mail or special delivery? Etc.

- You can use the abbreviation NTC for No-template control in line 213 as this may be more familiar to some readers.

- Line 215 please expand the word UCD.

- Line 216 a full stop is needed before this sentence “PCR…”.

- Line 240 expand upon first use.

- Line 241 “;” can be replaced with “.”.

-Line 244 is missing a full stop.

- Line 246 needs to be replaced to the following: “using Stata (16.0; StataCorp LLC, College Station, TX, US)”.

-Line 247 please cite the declaration of Helsinki.

- The consent for publication declaration is unnecessary.

- DSM-5 and other diagnostic criteria are mentioned in the consent form. It would be a good idea to mention them as inclusion criteria in the main manuscript.

- I would encourage the authors to be more detailed with the laboratory workflow.

Additionally, I recommend that the authors produce a graphical abstract outlining the workflow of the proposed study. This is not necessary per se; however, I believe it would strengthen the work and allow for a better graphical abstract representation of how the overall study will be conducted. Whether the authors decide to produce or not produce a graphical abstract does not personally affect my recommendation for publication.

Once again thank you for giving me the opportunity to read the present work. I recommend that the present work is published following these changes, and wish the authors the best of luck in conducting this study.

I acknowledge that I do not have any conflicts of interest. The present peer review is of my own expertise and artificial intelligence was not consulted.

Reviewer #2: Thank you for the opportunity to review this interesting paper. This manuscript presents a multicentre protocol for screening Fragile X disorders among Nigerian children with neurodevelopmental conditions, addressing an important evidence gap in sub-Saharan Africa. The manuscript is generally well structured but requires major revision to improve clarity, writing quality, and methodological presentation before publication.

In general, the manuscript should maintain a consistent protocol-writing style. The text occasionally shifts between protocol, grant, implementation, and advocacy language, particularly in the abstract, discussion, conclusion, follow-up, and timeline sections.

Under introduction

Maintain consistent terminology for Fragile X conditions throughout the manuscript. Standardize the use of terms such as Fragile X syndrome, Fragile X disorders, and FMR1-related disorders. Additionally, revise the sentence beginning "Despite global advances…" for grammar, style, and capitalization, removing the unnecessary capitalized "In."

Under objectives

Objective number 4, which stated, (To develop a context-appropriate model for integrating Fragile X screening into paediatric/developmental health services in Nigeria.)

I have noticed that the methods do not describe how the integration model will be developed. Every study objective should correspond to a defined methodology. Therefore, I suggest to describe the methodology for developing the model (e.g., stakeholder consultation, implementation evaluation, consensus methods) or revise the objective to reflect feasibility assessment.

Under methodology

Study Design: the statement: “This approach enables the study to track outcomes over time, assess intervention impacts…”The term “intervention impacts” may be misleading because this is primarily an observational screening protocol rather than an intervention trial. Therefore, replace with “assess longitudinal screening outcomes, referral processes, and implementation feasibility.”

Study Population: Children aged 1–18 years attending paediatric neurology/paediatric clinics. Briefly state how autism spectrum disorder, intellectual disability, and global developmental delay diagnoses are verified.

Sample Size: Sample size derived from records at a single hospital. Please justify the use of patient records from one coordinating centre to estimate the sample size for a multicentre national study. Discuss any limitations of this approach and add a brief rationale explaining why local records were used and acknowledge the absence of national prevalence data.

Sampling Technique: “This pragmatic sample is intended to estimate prevalence…” Clarify that prevalence estimates apply to the selected high-risk clinical cohort rather than the general paediatric population.

Statistical Analysis: “Descriptive statistics will be done…” Use standard statistical terminology throughout.Replace “will be done” with “will be performed” and “frequencies/percentages will be calculated.”

Statistical Analysis: “Logistic regression may be explored…”Clarify under what circumstances regression modelling will be performed (e.g., sufficient number of positive cases).

Result disclosure, counselling, and follow-up

In the manuscript, it is mentioned that targeted therapy will be offered using metformin for participants diagnosed with FXS. As written, this section could be interpreted as moving from screening into therapeutic intervention without adequate explanation. The wording should be revised carefully to avoid ambiguity and to maintain a protocol-appropriate scope. Therefore, I suggest to clearly distinguishing:

•what is part of the research protocol,

•what is part of post-test counselling,

•what constitutes standard clinical referral,

•and what, if any, treatment is outside the study but may be discussed or offered in routine care.

Under discussion

The discussion should focus on the anticipated contribution of the protocol rather than expected outcomes. Since this is a protocol paper, statements regarding improved clinical outcomes, national impact, or service transformation should be presented as potential benefits rather than established or expected results.

This is where the manuscript shifts most from a protocol to advocacy.

Examples include statements such as:

•the study "will substantially improve care"

•the project "will transform diagnosis and management"

•the programme "will improve the mental health and well-being of Nigerian children"

•statements implying that the screening programme will be successfully scaled nationally.

It is important to keep in mind that the discussion should acknowledge limitations more directly. These likely include:

•relatively small sample size,

•purposive rather than population-based recruitment,

•recruitment from selected health facilities,

•limited generalisability to all Nigerian children,

•possible underrepresentation of children outside formal care pathways,

•and feasibility challenges for counselling and follow-up.

Under Conclusion

The Conclusion reads more like the conclusion of a completed study than a study protocol. Rewrite with more focus on emphasize the anticipated contribution of the proposed study rather than implying demonstrated effectiveness or future implementation success.

Under Appendix

Ensure all appendices and supplementary materials are cited at their first mention in the manuscript. Several supporting documents (e.g., data collection proforma, examination checklists, workflow flowchart, SOP, and project timeline) are not consistently referenced.For example, CHAMP-FX Workflow Flowchart, It is listed at the end of the PDF but not cited in the manuscript. Please cite it in the Laboratory Workflow section

(S1 Appendix)

The statement : “Fragile X syndrome is a genetic condition caused by changes in the FMR1 gene on the X chromosome.” This is accurate, but may be difficult for some participants. Consider a plainer explanation such as “Fragile X syndrome is an inherited condition caused by a change in a gene.”

The word “premutation” may not be understandable to non-specialists. It should either be explained in plain language or omitted from the consent form if not essential for participant understanding.

**********

7. PLOS authors have the option to publish the peer review history of their article (what does this mean?). If published, this will include your full peer review and any attached files.

If you choose “no”, your identity will remain anonymous but your review may still be made public.

**Do you want your identity to be public for this peer review?**  For information about this choice, including consent withdrawal, please see our Privacy Policy.

Reviewer #1: No

Reviewer #2: No

**********

[NOTE: If reviewer comments were submitted as an attachment file, they will be attached to this email and accessible via the submission site. Please log into your account, locate the manuscript record, and check for the action link "View Attachments". If this link does not appear, there are no attachment files.]

To ensure your figures meet our technical requirements, please review our figure guidelines: https://journals.plos.org/plosone/s/figures

You may also use PLOS’s free figure tool, NAAS, to help you prepare publication quality figures: https://journals.plos.org/plosone/s/figures#loc-tools-for-figure-preparation.

NAAS will assess whether your figures meet our technical requirements by comparing each figure against our figure specifications.

Attachment Submitted filename: comments for PLOS ONE 2026.docx

# Author comment: Author response to Decision Letter 1

**DOI:** 10.1371/journal.pone.0355384.r003

15 Jul 2026

Response to Reviewer

We sincerely thank the editor and reviewers for the thorough review of our work and the constructive feedback, which has significantly strengthened our manuscript. We have carefully revised the manuscript to improve clarity, methodological rigour, consistency of terminology, and adherence to a protocol-writing style. Our detailed responses are provided below.

Editor

1.Editor comment: Please ensure that your manuscript meets PLOS ONE's style requirements, including those for file naming. The PLOS ONE style templates can be found at

https://journals.plos.org/plosone/s/file?id=wjVg/PLOSOne_formatting_sample_main_body.pdf and

https://journals.plos.org/plosone/s/file?id=ba62/PLOSOne_formatting_sample_title_authors_affiliations.pdf

Response: The corrections have been implemented throughout the manuscript in line with PLOS One guidelines.

2. Editor comment: Thank you for stating the following financial disclosure:

“This work was supported by the TETFund National Research Fund (NRF) [Grant No. TETFund/ES/DR&D-CE/NRF2024/SETI/HSW/00032/VOL.1]”

Please state what role the funders took in the study. If the funders had no role, please state: "The funders had no role in study design, data collection and analysis, decision to publish, or preparation of the manuscript."

Response: The statement has been added to the cover letter in line with the comment - "The funders had no role in study design, data collection and analysis, decision to publish, or preparation of the manuscript."

3. Editor comment: Thank you for stating the following in the Competing Interests section:

“Randi Hagerman (RH) reports research grants from Zynerba/Harmony for a cannabidiol (CBD) trial in Fragile X syndrome, and from Shionogi & Co., Ltd. for a zatolmilast study, awarded to UC Davis Medical Center.

RH also serves on advisory boards for Shionogi and Harmony Biosciences and is a member of the Clinical Trials Committee of the National Fragile X Foundation.

These relationships are unrelated to the current study and did not influence the study design, data collection, analysis, interpretation, or decision to publish.

All other authors declare no competing interests.”

We note that one or more of the authors are employed by a commercial company: “Zynerba/Harmony, Shionogi and Harmony Biosciences”.

1. Please provide an amended Funding Statement declaring this commercial affiliation, as well as a statement regarding the Role of Funders in your study. If the funding organization did not play a role in the study design, data collection and analysis, decision to publish, or preparation of the manuscript and only provided financial support in the form of authors' salaries and/or research materials, please review your statements relating to the author contributions, and ensure you have specifically and accurately indicated the role(s) that these authors had in your study. You can update author roles in the Author Contributions section of the online submission form.

Please also include the following statement within your amended Funding Statement.

“The funder provided support in the form of salaries for authors “R.H.” but did not have any additional role in the study design, data collection and analysis, decision to publish, or preparation of the manuscript. The specific roles of these authors are articulated in the ‘author contributions’ section.”

If your commercial affiliation did play a role in your study, please state and explain this role within your updated Funding Statement.

Response: Thank you for your comment. We wish to clarify that Prof. Randi Hagerman is not employed by Zynerba, Harmony Biosciences or Shionogi. She is employed by the University of California, Davis, USA. The competing interests statement refers to research grants awarded to UC Davis Medical Center for unrelated clinical trials and advisory board memberships. No commercial company funded the present study or provided salary support for any author. Accordingly, the requested statement regarding salary support from a commercial funder is not applicable. We clarify that the commercial entities had no role in the study design, data collection, analysis, interpretation, manuscript preparation or decision to publish.

3. Editor comment: Please also provide an updated Competing Interests Statement declaring this commercial affiliation along with any other relevant declarations relating to employment, consultancy, patents, products in development, or marketed products, etc.

Within your Competing Interests Statement, please confirm that this commercial affiliation does not alter your adherence to all PLOS ONE policies on sharing data and materials by including the following statement: "This does not alter our adherence to PLOS ONE policies on sharing data and materials.” (as detailed online in our guide for authors http://journals.plos.org/plosone/s/competing-interests) . If this adherence statement is not accurate and there are restrictions on sharing of data and/or materials, please state these. Please note that we cannot proceed with consideration of your article until this information has been declared.

Please include both an updated Funding Statement and Competing Interests Statement in your cover letter. We will change the online submission form on your behalf.

Response: Updated Funding Statement

Funding: This study was funded by the Tertiary Education Trust Fund (TETFund), National Research Fund (Grant No. TETFund/ES/DR&D-CE/NRF2024/SETI/HSW/00032/VOL.1). Prof. Randi Hagerman's unrelated institutional research at the University of California, Davis has received research funding from Zynerba/Harmony and Shionogi for Fragile X clinical trials. These commercial entities did not provide funding for the present study and had no role in the study design, data collection, data analysis, interpretation of the data, decision to publish, or preparation of the manuscript.

Updated Competing Interests Statement

Competing interests: Prof. Randi Hagerman reports unrelated institutional research grants awarded to the University of California, Davis from Zynerba/Harmony and Shionogi for Fragile X clinical trials. She also serves on advisory boards for Harmony Biosciences and Shionogi and is a member of the Clinical Trials Committee of the National Fragile X Foundation. These relationships are unrelated to the present study and did not influence the study design, data collection, analysis, interpretation, or decision to publish. All other authors declare no competing interests. This does not alter our adherence to PLOS ONE policies on sharing data and materials.

4. Editor comment: When completing the data availability statement of the submission form, you indicated that you will make your data available on acceptance. We strongly recommend all authors decide on a data sharing plan before acceptance, as the process can be lengthy and hold up publication timelines. Please note that, though access restrictions are acceptable now, your entire data will need to be made freely accessible if your manuscript is accepted for publication. This policy applies to all data except where public deposition would breach compliance with the protocol approved by your research ethics board. If you are unable to adhere to our open data policy, please kindly revise your statement to explain your reasoning and we will seek the editor's input on an exemption. Please be assured that, once you have provided your new statement, the assessment of your exemption will not hold up the peer review process.

Response: The Data Availability Statement has been modified to: No datasets were generated or analysed during the preparation of this study protocol. Following completion of the study, the de-identified dataset underlying the findings will be deposited in an appropriate publicly accessible data repository prior to publication, in accordance with the PLOS ONE Data Availability Policy, applicable ethical approvals, and participant confidentiality requirements.

5. Editor comment: Your ethics statement should only appear in the Methods section of your manuscript. If your ethics statement is written in any section besides the Methods, please move it to the Methods section and delete it from any other section. Please ensure that your ethics statement is included in your manuscript, as the ethics statement entered into the online submission form will not be published alongside your manuscript.

Response: The ethics statement has been removed from the abstract section of the manuscript.

6. Editor comment: If the reviewer comments include a recommendation to cite specific previously published works, please review and evaluate these publications to determine whether they are relevant and should be cited. There is no requirement to cite these works unless the editor has indicated otherwise.

Response: Thank you. The reviewer's comments do not include such a recommendation.

General comment

Reviewer comment: The manuscript occasionally shifts between protocol, grant, implementation and advocacy language.

Response: Thank you for this important observation. We have carefully revised the manuscript throughout to maintain a consistent protocol-writing style. Statements implying demonstrated effectiveness, service transformation or implementation success have been moderated to reflect the anticipated contribution of the proposed study. These revisions were made throughout the Abstract, Discussion, Conclusion and other relevant sections.

Introduction

Reviewer comment: Maintain consistent terminology for Fragile X conditions throughout the manuscript. Revise the sentence beginning "Despite global advances…"

Response: Thank you for this suggestion. We have standardised the terminology used throughout the manuscript to ensure consistency in the description of Fragile X disorders. The introductory section has also been revised for grammar, clarity and style, including the sentence beginning "Despite global advances…". These changes are reflected on Page 4, Lines 95–117.

Objectives

Reviewer comment: Objective 4 should correspond to a defined methodology.

Response: We appreciate this observation. We retained Objective 4 and added a corresponding methodology describing how the integration model will be developed. Specifically, the revised manuscript now describes stakeholder engagement involving the Child Neurology Society of Nigeria, Paediatric Association of Nigeria, the Federal Ministry of Health, selected State Ministries of Health, the National Primary Health Care Development Agency, and other relevant stakeholders. Findings from the study together with stakeholder recommendations will be synthesised to develop a context-appropriate model for integrating Fragile X screening into paediatric and developmental health services in Nigeria. These revisions are presented on Page 12, Lines 301–310.

Methodology

Study design

Reviewer comment: Replace "assess intervention impacts".

Response: Thank you. The wording has been revised to more accurately reflect the observational nature of the study. The manuscript now states that the study will assess longitudinal screening outcomes, referral processes and implementation outcomes rather than intervention impacts. These changes are reflected on Page 6, Lines 143–149.

Study population

Reviewer comment: Briefly state how autism spectrum disorder, intellectual disability and global developmental delay diagnoses are verified.

Response: We have expanded this section to describe the diagnostic procedures. Autism spectrum disorder, intellectual disability and global developmental delay are confirmed using DSM-5 diagnostic criteria through multidisciplinary assessments conducted by trained neurodevelopmental paediatricians, paediatric neurologists and a psychologist. We also describe the use of the Ages and Stages Questionnaire (ASQ-3) for children younger than four years and the Raven's Standard Progressive Matrices (Revised 2019) for children aged four years and above. These revisions appear on Pages 6–7, Lines 156–168.

Sample size

Reviewer comment: Justify the use of patient records from one coordinating centre.

Response: Thank you for this valuable comment. We have added a rationale explaining that, in the absence of national prevalence data on Fragile X syndrome among high-risk Nigerian children, patient records from the coordinating centre represented the best available local data for estimating the sample size. We also acknowledge the limitation of using a single-centre estimate while noting that the multicentre design enhances the representativeness of the study. These revisions are presented on Pages 7–8, Lines 181–194.

Sampling technique

Reviewer comment: Clarify that prevalence estimates apply to the selected high-risk cohort.

Response: The manuscript has been revised accordingly. It now clearly states that prevalence estimates apply to the selected high-risk clinical cohort rather than the general paediatric population. This clarification appears on Page 8, Lines 195–200.

Statistical analysis

Reviewer comment: Use standard statistical terminology.

Response: We have revised the statistical analysis section using standard statistical terminology. Phrases such as "will be done" have been replaced with "will be performed", and "frequencies and percentages will be calculated". These revisions are found on Pages 10–11, Lines 262–267.

Reviewer comment: Clarify under what circumstances logistic regression modelling will be performed.

Response: We have clarified that logistic regression analyses will only be performed if sufficient Fragile X-positive cases are available to support reliable multivariable modelling. Where the number of positive cases is insufficient, analyses will be limited to descriptive and bivariate methods. These revisions are presented on Page 11, Lines 268–272.

Result disclosure, counselling and follow-up

Reviewer comment: Clearly distinguish research procedures, post-test counselling, routine clinical referral and treatment outside the study.

Response: We appreciate this important observation. This section has been substantially revised to clearly distinguish activities that form part of the research protocol from routine clinical care. The revised manuscript now specifies that the research protocol includes result disclosure through post-test genetic counselling, while routine clinical management consists of referral to trained paediatric neurologists at participating centres, with referral to the coordinating centre where specialist input is required. Any treatment, including consideration of metformin where clinically indicated, will be provided as part of routine clinical care and is outside the scope of the research protocol. These revisions are presented on Pages 11–12, Lines 278–310.

Discussion

Reviewer comment: Focus the discussion on the anticipated contribution of the protocol and acknowledge study limitations.

Response: Thank you for this insightful suggestion. The Discussion has been extensively revised to maintain a protocol-focused perspective. Statements implying demonstrated effectiveness or future implementation success have been replaced with more appropriate language describing the anticipated contribution of the study. We have also expanded the limitations section to acknowledge the relatively small sample size, purposive sampling, recruitment from selected health facilities, limited generalisability, possible underrepresentation of children outside formal healthcare settings, and potential challenges related to counselling and long-term follow-up. These revisions are presented on Pages 13–14, Lines 337–357.

Conclusion

Reviewer comment: Rewrite the Conclusion to emphasise the anticipated contribution of the proposed study.

Response: The Conclusion has been revised to better reflect the purpose of a study protocol. It now emphasises the anticipated contribution of CHAMP-FX without implying demonstrated effectiveness or implementation success. These revisions are presented on Page 15, Lines 371–380.

Appendices

Reviewer comment: Ensure all appendices and supplementary materials are cited appropriately.

Response: We have reviewed the manuscript and ensured that all appendices and supplementary materials are cited at their first

Attachment Submitted filename: Response to Reviewer.docx

# Editor report: Decision Letter 1

**DOI:** 10.1371/journal.pone.0355384.r004

**Authors:** David Ikwuka

21 Jul 2026

Advancing Mental Health and Well-being of

Nigerian Children Through Public Health Screening for Fragile X Disorders (CHAMP-FX): Protocol of a prospective Multicentre Screening Study with Longitudinal Follow-up

PONE-D-26-20888R1

Dear Dr. Mbachu,

We’re pleased to inform you that your manuscript has been judged scientifically suitable for publication and will be formally accepted for publication once it meets all outstanding technical requirements.

Within one week, you’ll receive an e-mail detailing the required amendments. When these have been addressed, you’ll receive a formal acceptance letter and your manuscript will be scheduled for publication.

An invoice will be generated when your article is formally accepted. Please note, if your institution has a publishing partnership with PLOS and your article meets the relevant criteria, all or part of your publication costs will be covered. Please make sure your user information is up-to-date by logging into Editorial Manager at Editorial Manager® and clicking the ‘Update My Information' link at the top of the page. For questions related to billing, please contact billing support.

If your institution or institutions have a press office, please notify them about your upcoming paper to help maximize its impact. If they’ll be preparing press materials, please inform our press team as soon as possible -- no later than 48 hours after receiving the formal acceptance. Your manuscript will remain under strict press embargo until 2 pm Eastern Time on the date of publication. For more information, please contact onepress@plos.org.

Kind regards,

David Chibuike Ikwuka, Ph.D.

Academic Editor

PLOS One

Additional Editor Comments (optional):

Reviewers' comments:

# Editor report: Acceptance letter

**DOI:** 10.1371/journal.pone.0355384.r005

**Authors:** David Ikwuka

PONE-D-26-20888R1

PLOS One

Dear Dr. Mbachu,

I'm pleased to inform you that your manuscript has been deemed suitable for publication in PLOS One. Congratulations! Your manuscript is now being handed over to our production team.

At this stage, our production department will prepare your paper for publication. This includes ensuring the following:

* All references, tables, and figures are properly cited

* All relevant supporting information is included in the manuscript submission,

* There are no issues that prevent the paper from being properly typeset

You will receive further instructions from the production team, including instructions on how to review your proof when it is ready. Please keep in mind that we are working through a large volume of accepted articles, so please give us a few days to review your paper and let you know the next and final steps.

Lastly, if your institution or institutions have a press office, please let them know about your upcoming paper now to help maximize its impact. If they'll be preparing press materials, please inform our press team within the next 48 hours. Your manuscript will remain under strict press embargo until 2 pm Eastern Time on the date of publication. For more information, please contact onepress@plos.org.

You will receive an invoice from PLOS for your publication fee after your manuscript has reached the completed accept phase. If you receive an email requesting payment before acceptance or for any other service, this may be a phishing scheme. Learn how to identify phishing emails and protect your accounts at https://explore.plos.org/phishing.

If we can help with anything else, please email us at customercare@plos.org.

Thank you for submitting your work to PLOS ONE and supporting open access.

Kind regards,

PLOS ONE Editorial Office Staff

on behalf of

Dr David Chibuike Ikwuka

Academic Editor

PLOS One
